# Attenuated Getah virus confers protection against multiple arthritogenic alphaviruses

**DOI:** 10.1371/journal.ppat.1012700

**Published:** 2024-11-18

**Authors:** Zhiwen Jiang, Andres Merits, Ying Qin, Gang Xing, Letian Zhang, Jie Chen, Ningning Wang, Margus Varjak, Xiaofeng Zhai, Dongyan Li, Wanjie Song, Shuo Su

**Affiliations:** 1 Sanya Institute of Nanjing Agricultural University, Academy for Advanced Interdisciplinary Studies, Jiangsu Engineering Laboratory of Animal Immunology, Institute of Immunology and College of Veterinary Medicine, Nanjing Agricultural University, Nanjing, China; 2 Institute of Bioengineering, University of Tartu, Tartu, Estonia; 3 MOA Key Laboratory of Animal Virology, Zhejiang University, Hangzhou, China; 4 Institute of Technology, University of Tartu, Tartu, Estonia; National Institute of Allergy and Infectious Diseases Laboratory of Viral Diseases, UNITED STATES OF AMERICA

## Abstract

Alphaviruses are important arthropod-transmitted pathogens of humans and livestock. Getah virus (GETV) is an arthritogenic alphavirus that causes disease in horses and piglets; it also poses a potential threat to humans. A live attenuated vaccine candidate named GETV-3ΔS2-CM1, harbouring a deletion in nonstructural protein 3 and substitutions in the capsid protein, is genetically stable and exhibits robust immunogenicity. It was shown to confer passive protection to piglets born to immunized sows. In mice, a single dose of GETV-3ΔS2-CM1 protected against infection with different strains of GETV, Semliki Forest virus, Ross River virus, o’nyong’nyong virus, chikungunya virus, and Barmah Forest virus. Chimaeras based on the GETV-3ΔS2-CM1 backbone maintained both the attenuated phenotype and high immunogenicity. The safety, efficacy, and ability to induce protection against multiple alphaviruses highlights the potential of GETV-3ΔS2-CM1 and chimaeras using this backbone as promising vaccine candidates. By contributing simultaneously to the wellbeing of animals and humans, our universal next generation vaccine strategy helps to achieve "One Health" goals.

## Author summary

GETV is a zoonotic mosquito transmitted alphavirus. It is causing infections of livestock and has a potential to harm humans. In the present study, we developed an attenuated variant of GETV. Immunization with this vaccine candidate results in antibody response that protects mice, pigs, and the progeny of immunized mothers against challenge with high dose of GETV. Importantly, the immunization also protects against the challenge with other arthritogenic alphaviruses. Furthermore, we demonstrate that attenuated GETV backbone can be used for construction of chimeric alphaviruses that represent promising vaccine candidates. This study highlights the applicability of attenuated GETV for the prevention and control of multiple arthritogenic alphaviruses in animals under the "One Health" principle.

## Introduction

Alphaviruses (family *Togaviridae*) are arthropod-transmitted pathogens. Based on the symptoms of infection, they are classified as arthritogenic or encephalitic viruses. Although many alphaviruses are currently endemic only in specific regions, they are known to extend their geographical distribution; in recent decades, chikungunya virus (CHIKV) has caused massive outbreaks [1], spurring advancements in the development of effective antiviral approaches.

In preclinical trials, vaccine candidates, including nucleic acids, virus-like particles (VLPs), and inactivated, live attenuated and recombinant viruses, have been shown to be effective against alphaviruses [2]. Notably, a live attenuated vaccine (LAV) against CHIKV (Ixchiq; formerly known as VLA1553 or Δ5nsP3 [3]) recently received FDA approval [4]. LAVs have the advantage of high immunogenicity, in some cases from a single dose; however, their safety needs to be carefully examined. Thus, concerns regarding pathogenicity and/or reversions have prevented or restricted the use of previously developed vaccines against CHIKV and Venezuelan equine encephalitis virus (VEEV) [5,6].

Getah virus (GETV) has been detected in various wild animal species, and outbreaks of this virus in domestic animals have been reported in Asian countries [7–9]. In newborn piglets, GETV infection causes diarrhoea, hind limb paralysis, depression and severe cases leading to fatality are common. In pregnant sows, GETV infection can result in abortion as well as vertical transmission to the offspring [10]. Early serologic surveillance has also revealed the presence of neutralizing antibodies against GETV in humans [11,12].

**Fig 1:**
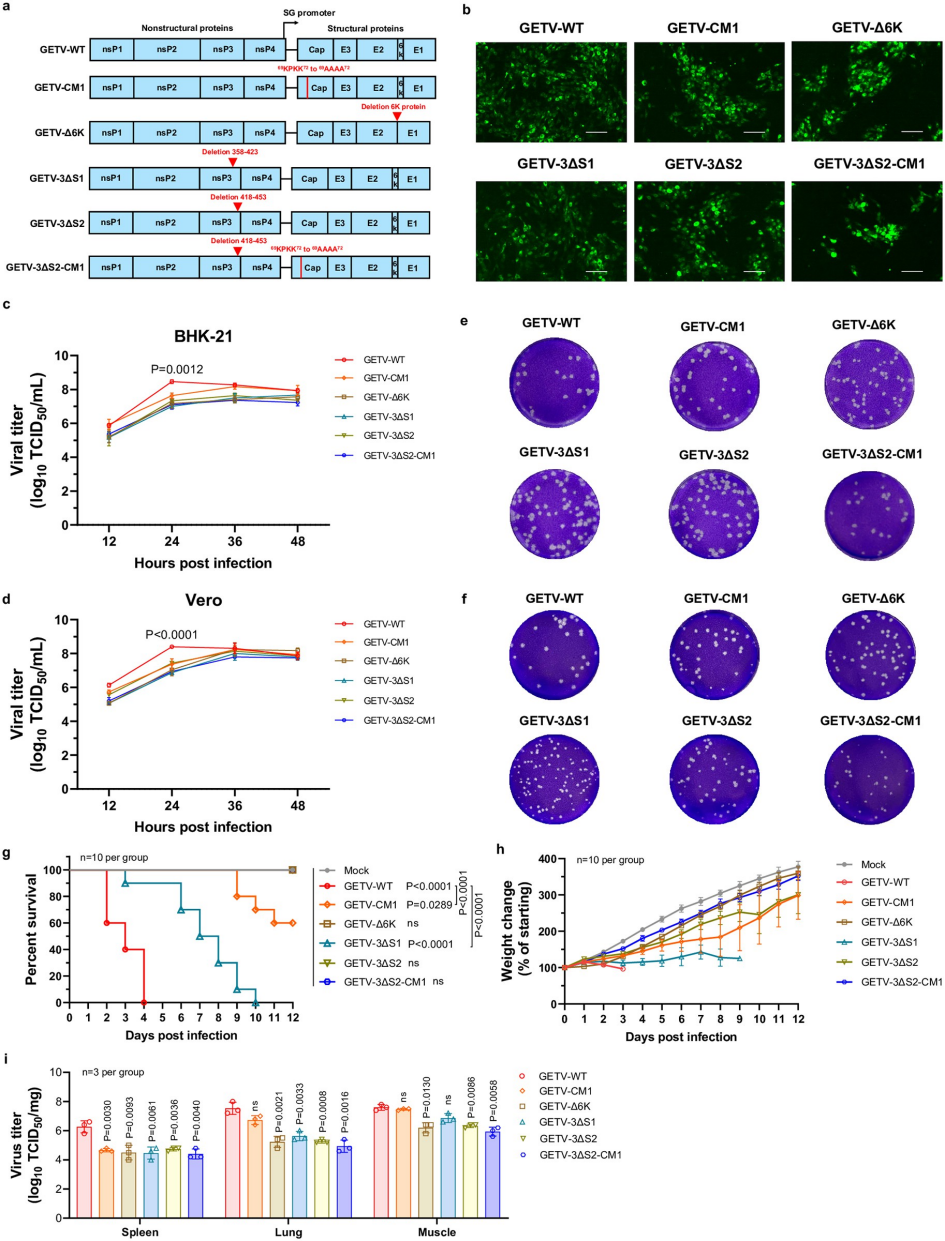
GETV-3ΔS2, GETV-Δ6K and GETV-3ΔS2-CM1 are attenuated in vitro and are avirulent in suckling mice. **(a)** Schematic representation of the genomes of the constructed viable mutant viruses. Red triangles and solid lines point to the introduced mutations. **(b)** BHK-21 cells infected with WT GETV or its mutants were fixed at 12 hpi. GETV infection was visualized by IFA using an antibody against E1 of GETV. Images were acquired with a Nikon microscope; scale bar, 500 μM. **(c, d)** Multistep growth curves of WT GETV, GETV-CM1, GETV-Δ6K, GETV-3ΔS1, GETV-3ΔS2, and GETV-3ΔS2-CM1 on BHK-21 **(c)** and Vero **(d)** cells. Cells were infected at a MOI of 0.01. Due to the compact format, some error bars cannot be shown. P values indicating statistical significance of differences between titres of WT GETV and GETV-3ΔS2-CM1 measured in three independent experiments at 24 hpi are shown. **(e, f)** Plaque morphologies of the above listed viruses on BHK-21 **(e)** and Vero **(f)** cell monolayers. The cells were fixed and stained on Day 2 (BHK-21) or Day 3 (Vero) post infection. **(g, h)** Two-day-old mice (n = 10 per group) were subcutaneously inoculated with 3×10^5^ TCID_50_ of WT GETV, GETV-CM1, GETV-Δ6K, GETV-3ΔS1, GETV-3ΔS2, or GETV-3ΔS2-CM1 or mock-infected with PBS. Survival **(g)** and weight changes **(h)** were monitored daily. **(i)** Mice (n = 3 per group) were infected as described above; on Day 2 post infection, the animals were sacrificed, and the viral loads in the spleen, lung and muscle tissues were measured by TCID_50_ assays. For panel **g** statistical analysis was performed using the log-rank test. P-values indicating statistical significance are shown as follows: the first values listed are in comparison to Mock; other values represent comparison of GETV-CM1 and GETV-3ΔS1 infected mice to WT GETV infected mice. For panels **c**, **d** and **i** statistical analysis was performed using Student’s t test. On panel **i** P-values indicate statistical significance of differences between viral loads in tissues of WT GETV and mutant virus infected mice; ns, not significant.

GETV belongs to the Semliki Forest virus (SFV) antigenic complex, which also includes major human pathogens such as CHIKV, o’nyong’nyong virus (ONNV), Ross River virus (RRV), and Mayaro virus (MAYV) (https://ictv.global/report/chapter/togaviridae/togaviridae). It has a positive-sense RNA genome approximately 11.5 kb in length that harbours two open reading frames (ORFs) [13]. ORF1 encodes precursors for nonstructural proteins 1–4 (nsP1-4), which are subunits of the viral RNA replicase, while ORF2 encodes precursors of capsid protein (Cap) and envelope proteins (Fig 1a). Only a few attempts to develop vaccines against GETV have been reported, including dual inactivated vaccines targeting GETV and Japanese encephalitis virus (JEV) developed by Nisseiken (Japan). However, outbreaks of GETV still occur in vaccinated areas [14]. This can primarily be attributed to the insufficient immunogenicity of the inactivated vaccine. Consistently, mice immunized with this vaccine exhibited low antibody titres [15]. Thus, developing an effective and safe vaccine to prevent future epidemics of GETV in livestock—and potentially in humans—remains important.

Here, we utilized an infectious cDNA (icDNA) clone of the GETV-HN strain for the rational design of a live attenuated GETV vaccine candidate, GETV-3ΔS2-CM1. The virus was strongly attenuated in neonatal mice, exhibited robust immunogenicity in 3- and 6-week-old ICR mice, and was safe in pregnant sows and piglets. Passive immunity acquired through colostrum feeding protected piglets from high-dose GETV challenge. A single immunization of mice with GETV-3ΔS2-CM1 provided protection against viremia induced by subsequent infection with RRV, SFV, ONNV, and the distantly related Barmah Forest virus (BFV); importantly, chimeric viruses based on the GETV-3ΔS2-CM1 backbone maintained these favourable properties. Collectively, these findings underscore the potential of GETV-3ΔS2-CM1 as an efficacious vaccine platform for combating arthritogenic alphavirus infections, restricting their spread and preventing potential spill-over events.

## Results

### Mutations in the HVD of nsP3 and capsid protein reduce GETV pathogenicity in mice

Previous studies have revealed that alphaviruses can be attenuated by deletions in the hypervariable domain (HVD) of nsP3, mutations in capsid protein regions and deletions of the 6K region [3,16,17]. Using an infectious clone of GETV-HN, six mutant GETV genomes were constructed (Fig 1a and S1a Fig). Mutations introduced into the nsP3 region removed proline-rich sequences; analogous regions of other alphaviruses have been shown to interact with host factors, including CD2AP [18,19]. Consistently, when the same mutations were introduced into a GETV nsP3 expression vector, co-transfection experiments revealed that 3ΔS1+2 entirely abolished the interaction and colocalization of GETV nsP3 with mouse CD2AP as well as with BIN1 (S1b–S1d Fig). Separately, the 3ΔS1 and 3ΔS2 mutations decreased the ability of nsP3 to interact with CD2AP and 3ΔS1 mutation also slightly decreased the ability of nsP3 to interact with BIN1 (S1b and S1c Fig). Notably, we did not detect a significant impact of 3ΔS2 on the interaction of GETV nsP3 with other cellular proteins known to interact with the HVD of alphaviruses [19], including CAPZA, CAPZB, PARP1, RPL6, RPL7A or RPL24 (S2 Fig). In the structural region of GETV, we substituted residues 69–72 of the capsid protein (KPKK in GETV-HN) with alanine residues (CM1) or removed the 6K region (Δ6K). The former mutation was predicted to alter the subcellular localization of the GETV capsid protein; indeed, the substitutions resulted in more diffused localization of capsid protein in the nucleus suggesting partial loss of its nucleolar localization (S1e Fig). Finally, we combined the second deletion in nsP3 with substitutions in the capsid protein (3ΔS2-CM1).

Virus harbouring the 3ΔS1+2 mutation could not be rescued. In contrast, all other mutant viruses were viable (Fig 1b). In BHK-21 and Vero cells, the mutant viruses exhibited a growth delay but, nevertheless, reached final titres comparable to those of WT GETV (Fig 1c and 1d). However, compared to WT GETV, mutant viruses generally produced smaller plaques (Fig 1e and 1f). This effect was especially pronounced for GETV-3ΔS2-CM1 in Vero cells (Fig 1f). In NIH-3T3 cells a prominent reduction of GETV-3ΔS2-CM1 replication, viral RNA synthesis and release were observed (S3a–S3c Fig). These effects were unlikely caused by type I interferons (IFN) as, compared to WT GETV, GETV-3ΔS2-CM1 infection resulted in much lower levels of induction of IFN-β mRNA expression (S3d Fig). Thus, mutations in GETV-3ΔS2-CM1 attenuate its replication possibly by altering the localization of the capsid protein inside the nucleus of infected cells as well as through modulation of the interaction between nsP3 and CD2AP. Prominent attenuation requires a combination of both of these mutations.

In a lethal suckling mouse model, all mutant viruses displayed attenuated phenotypes compared to those of WT GETV. However, all mice infected with GETV-3ΔS1 and some of the mice infected with GETV-CM1 ultimately succumbed to the infection, while all of the mice infected with the other three mutant viruses survived (Fig 1g) and did not display a significant decrease in weight gain (Fig 1h). Analysis of the viral load in organs on Day 2 post infection revealed that the titres of all five mutant viruses were similarly reduced in the spleen. In the lung and muscle tissues, the virulent mutants (GETV-3ΔS1 and GETV-CM1) displayed a smaller reduction in titre than the avirulent mutants (Fig 1i). Based on these data, avirulent GETV-3ΔS2-CM1 was selected for subsequent analysis.

### GETV-3ΔS2-CM1 is genetically stable and induces protective immunity in a mouse model

The genetic stability of GETV-3ΔS2-CM1 was analysed in vitro and in vivo. Five consecutive low multiplicity of infection (MOI = 0.01) passages of GETV-3ΔS2-CM1 in BHK-21 cells did not lead to reversion of the introduced changes. The same was observed when the virus was serially passaged in neonatal mice. When the fifth-passage stocks derived from in vitro or in vivo experiments were tested in a neonatal mouse model, no discernible alterations in virulence or viral titres in different organs were observed (S4 Fig). Thus, GETV-3ΔS2-CM1 remains avirulent in neonatal mice, indicating also a lack of second-site adaptive mutations.

**Fig 2:**
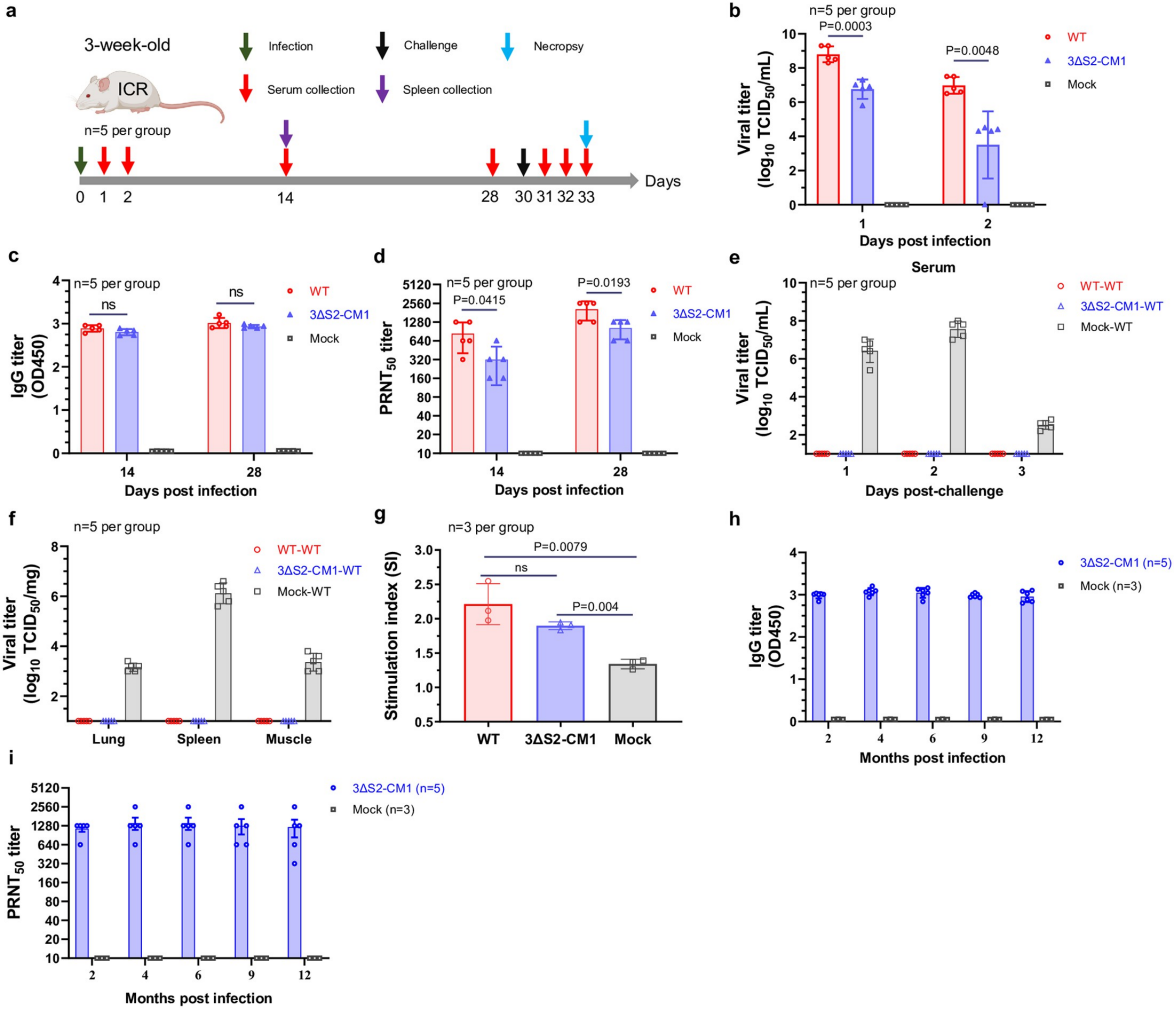
Immune response generated by infection with GETV-3ΔS2-CM1 protects mice against subsequent WT GETV challenge. **(a)** Schematic description of the experiment. Three-week-old ICR mice were infected with 1 × 10^5^ TCID_50_ of WT GETV or GETV-3ΔS2-CM1. Antibody responses were measured at 14 and 28 days post infection. At 30 days post infection, the mice were subcutaneously challenged with 1 × 10^6^ TCID_50_ of WT GETV. Serum samples and organs were collected at the indicated time points. **(b)** Viral titres in the sera of the WT GETV- and GETV-3ΔS2-CM1-infected mice (n = 5) at 1 and 2 days post infection were determined using a TCID_50_ assay. **(c)** GETV-specific IgG titres in serum at 14 and 28 days post infection measured by ELISA performed using recombinant GETV p62-E1 protein and presented as OD450 values. **(d)** Neutralizing antibody titres (PRNT_50_) in sera at 14 and 28 days post infection determined using WT GETV virions. **(e)** Viremia on Day 1 to Day 3 post-challenge. **(f)** Viral loads in lung, spleen, and muscle tissues on Day 3 post-challenge. **(g)** Specific splenocyte proliferation. Two weeks after immunization, mouse spleens (n = 3 per group) were collected, spleen cells were isolated and restimulated with recombinant GETV p62-E1 protein. Cell proliferation was measured using a CCK-8 assay. The splenocyte stimulation index was calculated using the formula SI = (OD stimulant—OD 1640)/(OD control—OD 1640). **(h, i)** Three-week-old ICR mice were infected with 1 × 10^5^ TCID_50_ of GETV-3ΔS2-CM1 (n = 5) or mock infected (n = 3). Serum samples were collected at 2, 4, 6, 9, and 12 months after infection. **(h)** Anti-GETV IgG titres were measured as described for (c). **(i)** Neutralizing antibody titres were measured as described for (d). For panels **b**, **c**, **d**, and **g** statistical analysis was performed using Student’s t test. P-values are shown; ns, not significant. *Panel a in this figure was created with BioRender*.

Three-week-old mice were infected with WT GETV or GETV-3ΔS2-CM1 (Fig 2a). As expected, GETV-3ΔS2-CM1-infected mice exhibited reduced viremia compared to that of mice infected with WT GETV (Fig 2b). Nevertheless, at 14 and 28 days post infection, the GETV-3ΔS2-CM1-infected mice displayed robust seroconversion; the titres of GETV-specific IgG (Fig 2c) and neutralizing antibodies (Fig 2d) were only slightly lower than those elicited by the WT GETV. IgG isotype analysis revealed that infection with both of WT GETV and GETV-3ΔS2-CM1 induced predominantly IgG2 isotypes (S5a and S5b Fig). The IgG2a to IgG1 ratio in the WT GETV group was approximately 1 while in the GETV-3ΔS2-CM1 groups it was greater than 1 (S5c Fig) suggesting a slightly Th1-biased immune response. Notably, upon challenge with high dose of WT GETV, no mice immunized with either WT GETV or GETV-3ΔS2-CM1 showed detectable viremia (Fig 2e). The examination of autopsied organs on Day 3 post-challenge revealed no infectious virus in the immunized groups (Fig 2f). Furthermore, compared to spleen cells from mock-immunized animals, spleen cells derived from GETV-3ΔS2-CM1-immunized mice exhibited enhanced in vitro proliferation upon stimulation with recombinant GETV p62-E1 protein (Fig 2g). This finding suggested that, akin to other LAVs, GETV-3ΔS2-CM1 also elicits a cellular immune response. To evaluate the duration of the immune response, 3-week-old mice were subcutaneously vaccinated with GETV-3ΔS2-CM1, and sera were collected at 2, 4, 6, 9, and 12 months after immunization. GETV-3ΔS2-CM1 induced robust and long-lasting increases in the levels of GETV-specific IgG (Fig 2h) and neutralizing antibodies (Fig 2i).

To assess the protective efficacy of immunization with GETV-3ΔS2-CM1 against heterologous GETV strains, 3-week-old mice were immunized with GETV-3ΔS2-CM1 and 14 days later animals were challenged with high doses of homologous (GETV-HN) and heterologous (GETV-GX and GETV-FJ) GETV strains. Vaccinated mice exhibited complete protection against all used GETV strains (HN, GX, and FJ strain) (S6a Fig). As expected, all GETV-3ΔS2-CM1 immunized mice had high titers of neutralizing antibodies; importantly, these were not significantly increased by the challenge with any GETV strain (S6b Fig). The lack of anamnestic response confirms that a single vaccination with GETV-3ΔS2-CM1 resulted in sterilizing immunity.

**Fig 3:**
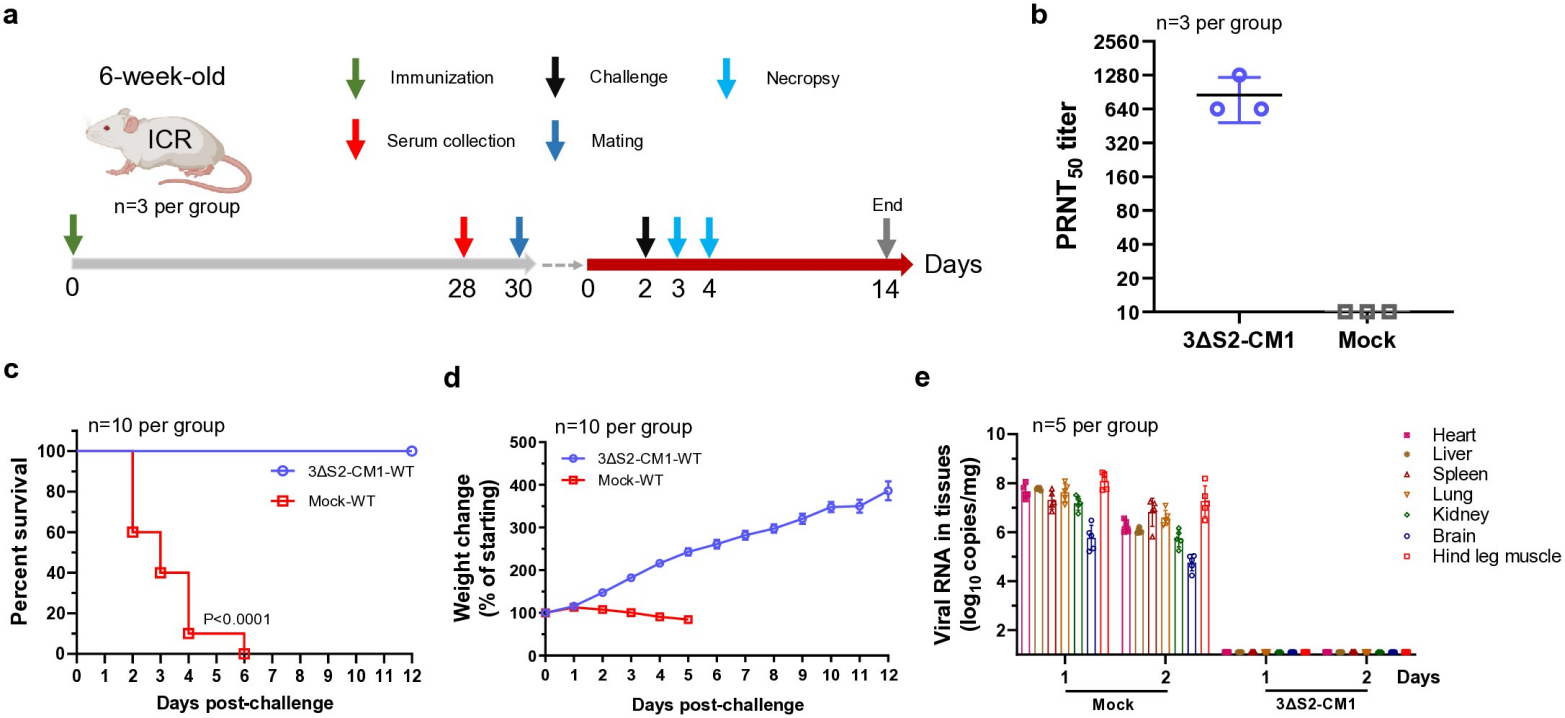
Immunization with GETV-3ΔS2-CM1 generates passive immunity that protects neonatal mice against WT GETV challenge. **(a)** Schematic description of the experiment. Six-week-old female ICR mice were immunized with 1 × 10^5^ TCID_50_ of GETV-3ΔS2-CM1 or mock-immunized (n = 3 per group). At 28 days postimmunization, neutralizing antibody titres in the serum samples were measured by the PRNT assay **(b)**, and then, mating was performed. Two-day-old mice from immunized and mock-immunized mothers (n = 10 per group) were challenged with 3 × 10^5^ TCID_50_ of WT GETV, and survival **(c)** and weight changes **(d)** were monitored for 12 days. **(e)** Two-day-old mice from immunized and mock-immunized mothers (n = 10 per group) were challenged with 3 × 10^5^ TCID_50_ of WT GETV. On Day 1 and Day 2 post-challenge, five mice per group were sacrificed, and viral RNA loads in the indicated organs were measured using RT-qPCR. For panel **c** statistical analysis was performed using the log-rank test. *Panel a in this figure was created with BioRender*.

Next, we analysed whether immunity acquired from GETV-3ΔS2-CM1-immunized mothers could protect neonates against WT GETV infection (Fig 3a). As expected, immunization of 6-week-old female mice with GETV-3ΔS2-CM1 resulted in high titres of neutralizing antibodies (Fig 3b). When 2-day-old mice born to immunized mothers were challenged with WT GETV, they exhibited complete protection; in contrast, mice from the control group experienced rapid deterioration and succumbed to the infection (Fig 3c and 3d). No viral genomes were detected in the heart, liver, spleen, lung, kidney, brain or leg muscle of mice born to GETV-3ΔS2-CM1-immunized mothers and infected with WT GETV. In contrast, high levels of GETV RNA were detected in the organs of animals from the control group on Days 1 and 2 post-challenge (Fig 3e). Thus, maternal antibodies, which are transferred to pups through the placenta and milk, protect neonates against WT GETV infection.

### GETV-3ΔS2-CM1 is avirulent in piglets

**Fig 4:**
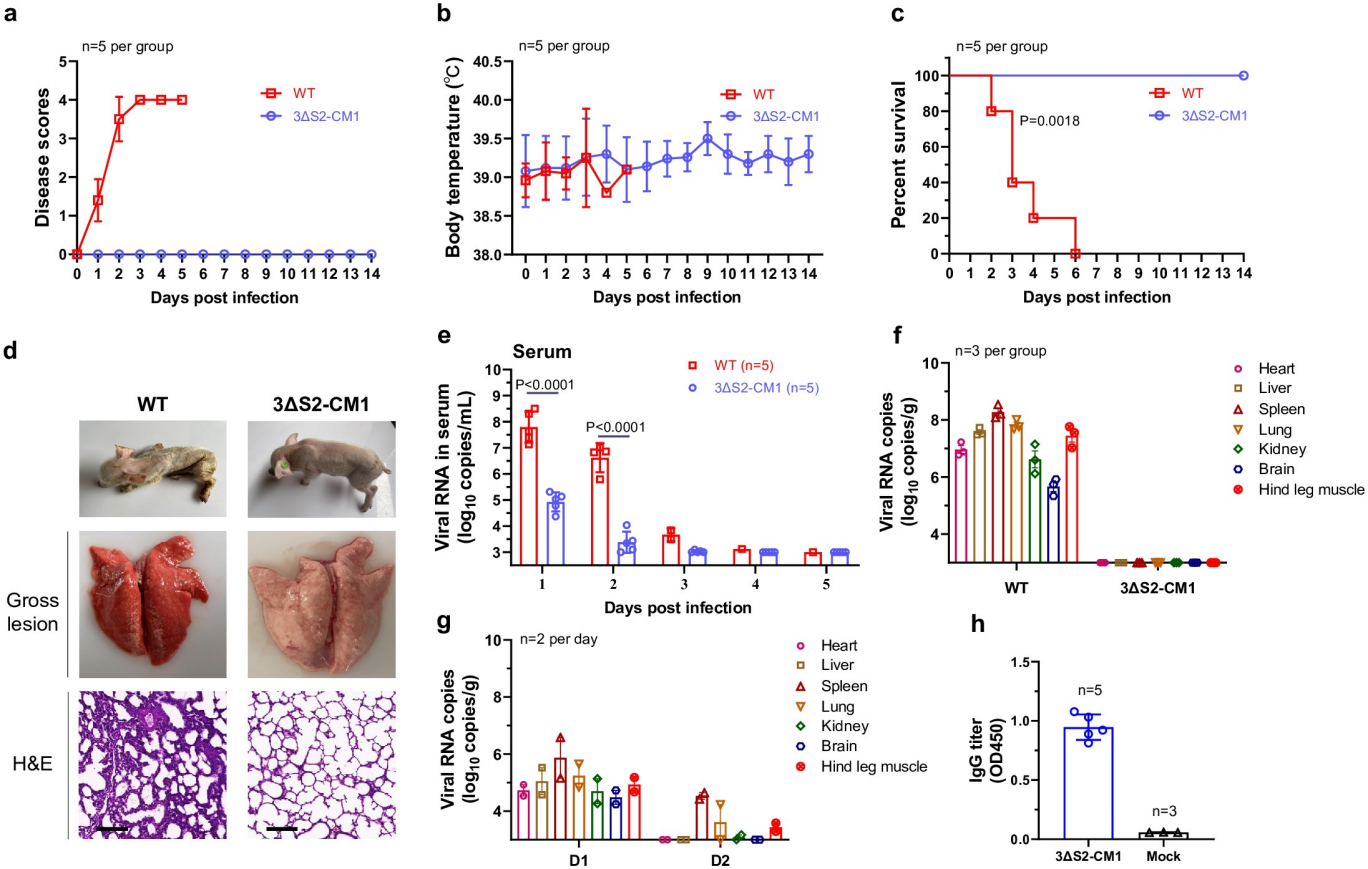
GETV-3ΔS2-CM1 is attenuated in newborn piglets. One-day-old piglets not fed colostrum were infected with 5 × 10^7^ TCID_50_ of WT GETV or GETV-3ΔS2-CM1 (n = 5 per group). Disease scores were monitored for 14 days; sera from surviving animals were collected on Day 14. **(a)** The disease scores are based on the presence or absence of diarrhoea, hind limb paralysis and depression. Piglets without signs of disease were scored as 0. **(b)** Body temperature and **(c)** survival of infected piglets. **(d)** Examples of the clinical manifestations of disease in piglets; pathological changes in the lungs and H&E staining (scale bar 200 μm) of lung tissues from WT GETV-infected piglets that reached the clinical endpoint are shown. GETV-3ΔS2-CM1-infected piglets were from an additional group; these animals were sacrificed on Day 3 post infection. **(e)** Viral RNA levels in sera from infected piglets. **(f)** Piglets (n = 3 per group) were infected with 5 × 10^7^ TCID_50_ of WT GETV or GETV-3ΔS2-CM1 and euthanized on Days 2 and 3 post infection (WT GETV group) or on Day 3 post infection (GETV-3ΔS2-CM1 group). Viral RNA levels in different organs were measured using RT-qPCR. **(g)** A group of 1-day-old piglets (n = 4) was infected with GETV-3ΔS2-CM1; two animals were euthanized on Day 1, and two animals were euthanized on Day 2 post infection, after which autopsy was performed, and viral RNA loads in different organs were determined using RT-qPCR. **(h)** Anti-GETV IgG titres in GETV-3ΔS2-CM1-infected animals (n = 5) at 14 days post infection. Mock: sera from mock-infected animals (n = 3). For panel **e** statistical analysis was performed using Student’s t test. P-values are shown. For panel **c** statistical analysis was performed using the log-rank test.

For this study, we developed a novel sensitive model of lethal GETV infection in newborn piglets. Using this model, we compared the virulence of WT GETV and that of GETV-3ΔS2-CM1 using 1-day-old colostrum-deprived piglets. Piglets infected with a high dose of WT GETV exhibited severe diarrhoea, hind limb paralysis, tongue tremors, mental depression, and other disease signs but not a fever (Fig 4a and 4b). No adverse reactions were observed for piglets infected with GETV-3ΔS2-CM1 and none of the animals died. In contrast, all of the piglets infected with WT GETV reached the clinical endpoint by Day 6 post infection (Fig 4c). Autopsy of piglets in the WT GETV group revealed severe haemorrhage in the lungs and haemocyte infiltration in H&E-stained tissue sections; these disease signs were not detected in GETV-3ΔS2-CM1-infected piglets (Fig 4d). Furthermore, the number of viral RNA copies in the serum of piglets infected with WT GETV was significantly greater than that in the serum of GETV-3ΔS2-CM1-infected animals (Fig 4e). Similarly, viral RNA copy numbers were high in all organs of animals infected with WT GETV; in contrast, in the GETV-3ΔS2-CM1 group, viral RNA levels were under the detection limit at Day 3 post infection (Fig 4f). In another experiment, we observed GETV-3ΔS2-CM1 RNA levels slightly above the limit of detection in all analysed organs on Day 1 post infection; on Day 2 post infection, viral RNA was detected only in the spleen, lung, and hind leg muscle (Fig 4g) indicating reduced in vivo dissemination and rapid clearance of GETV-3ΔS2-CM1. Finally, analysis of anti-GETV IgG titres in sera collected from GETV-3ΔS2-CM1-infected piglets at 14 days post infection revealed complete seroconversion (Fig 4h). The measured IgG titres were somewhat lower than these in mice sera (compare Figs 4h and 2c), possibly reflecting differences of GETV-3ΔS2-CM1 infection and/or immune responses in different species. Taken together, these experiments demonstrated that GETV-3ΔS2-CM1 is avirulent for piglets; however, it is capable of inducing a potent antibody response.

### The vaccination of pregnant sows with GETV-3ΔS2-CM1 is safe and results in the passive protection of newborn piglets

**Fig 5:**
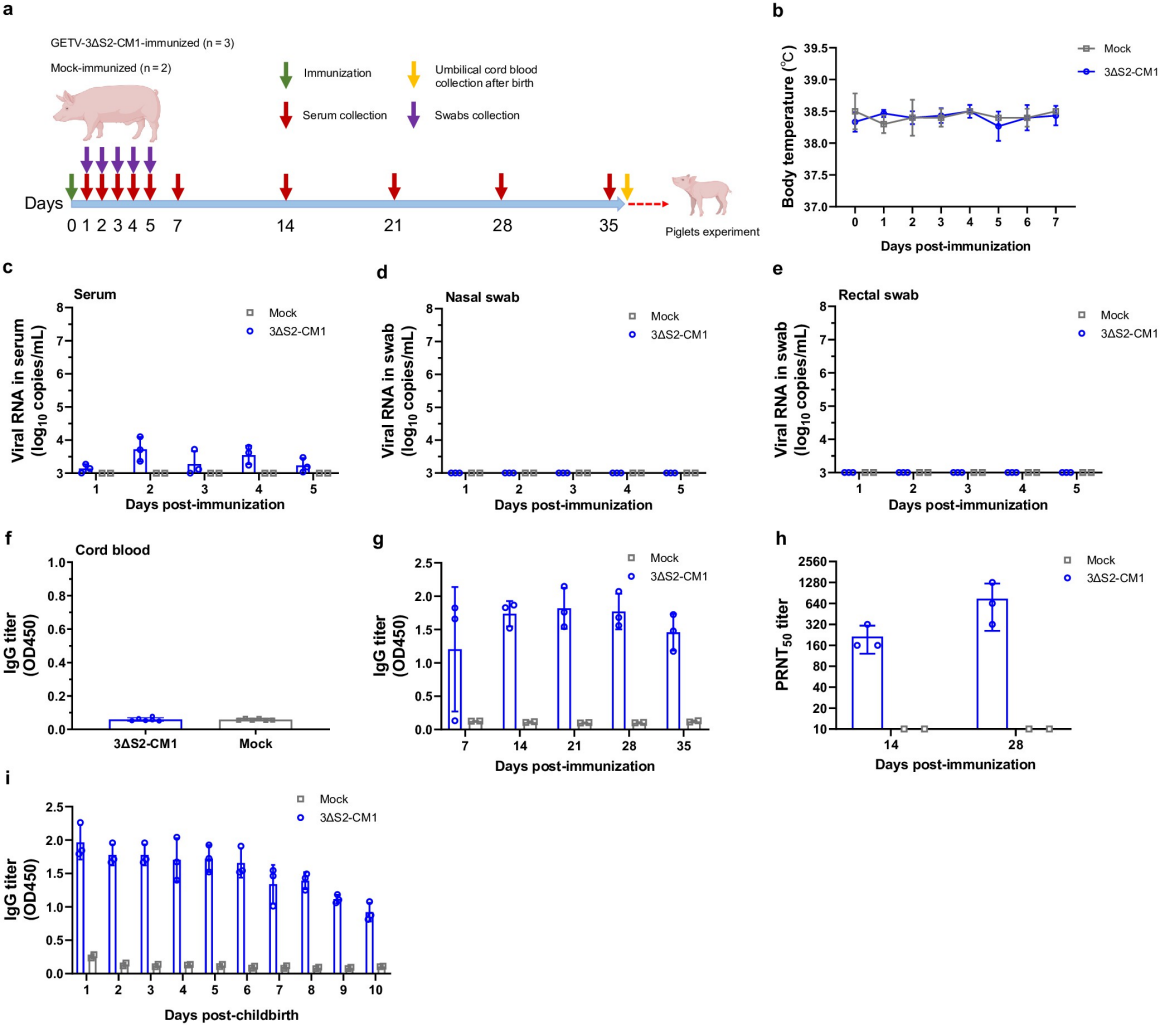
GETV-3ΔS2-CM1 is safe and immunogenic in pregnant sows. **(a)** Schematic description of the experiment. Pregnant sows were intramuscularly immunized with 1 × 10^5^ TCID_50_ of GETV-3ΔS2-CM1 (n = 3) or mock-immunized (n = 2). Serum samples, nasal swabs, and rectal swabs were collected at the indicated time points. **(b)** Body temperatures of sows within 1 week after immunization. **(c-e)**. Viral RNA copy numbers in the serum **(c)**, nasal swabs **(d)**, and rectal swabs **(e)** at 1–5 days postimmunization. **(f)** GETV-specific IgG titres in the cord blood of newborn piglets from immunized and mock-immunized sows and **(g)** in sera from sows at 1–5 weeks postimmunization were measured by ELISA. **(h)** Neutralizing antibody titres in the sera from sows at Days 14 and 28 postimmunization were measured by PRNT. **(i)** Titres of GETV-specific IgG antibodies in the maternal milk at 1–10 days after natural parturition were measured by ELISA. For **f, g**, and **i** the GETV-specific IgG titres are presented as OD450 values. *Panel a in this figure was created with BioRender*.

To evaluate the immunogenicity of GETV-3ΔS2-CM1 in pregnant sows, animals with an expected parturition in approximately 35 days were intramuscularly immunized with 1 × 10^5^ TCID_50_ of GETV-3ΔS2-CM1 (Fig 5a). The body temperature of the immunized animals, monitored over a period of 7 days, was similar to that of the mock-immunized animals (Fig 5b). The amount of infectious virus in the serum samples collected from 1 to 5 days postimmunization was below the detection limit of TCID_50_ assay. Consistently, only trace amounts of GETV RNA were detected in serum samples (Fig 5c), and GETV RNA was undetectable in nasal or rectal swabs (Fig 5d and 5e). The immunized sows gave birth without any miscarriages, and no abnormalities, such as depression or not eating, were observed. GETV RNA was undetectable in the umbilical cord and foetal membranes collected from the sows at the time of parturition indicating that unlike WT GETV [10] attenuated GETV-3ΔS2-CM1 lacks a vertical transmission. Consistent with the lack of transplacental transfer of antibodies in pigs [20] no IgG antibodies against GETV p62-E1 were detected in the cord blood of newborn piglets (Fig 5f). Taken together, these data confirm that GETV-3ΔS2-CM1 has a high safety profile in pregnant sows. At the same time, immunization with GETV-3ΔS2-CM1 resulted in a strong immune response: GETV-specific antibodies were detected in the serum as early as one week after immunization, and their titres had increased by week 2 and remained high during the 5-week monitoring period (Fig 5g). The sera from immunized animals neutralized GETV (Fig 5h) and, after natural parturition, anti-GETV antibodies were present in the milk of immunized sows for the entire monitoring period of 10 days (Fig 5i).

**Fig 6:**
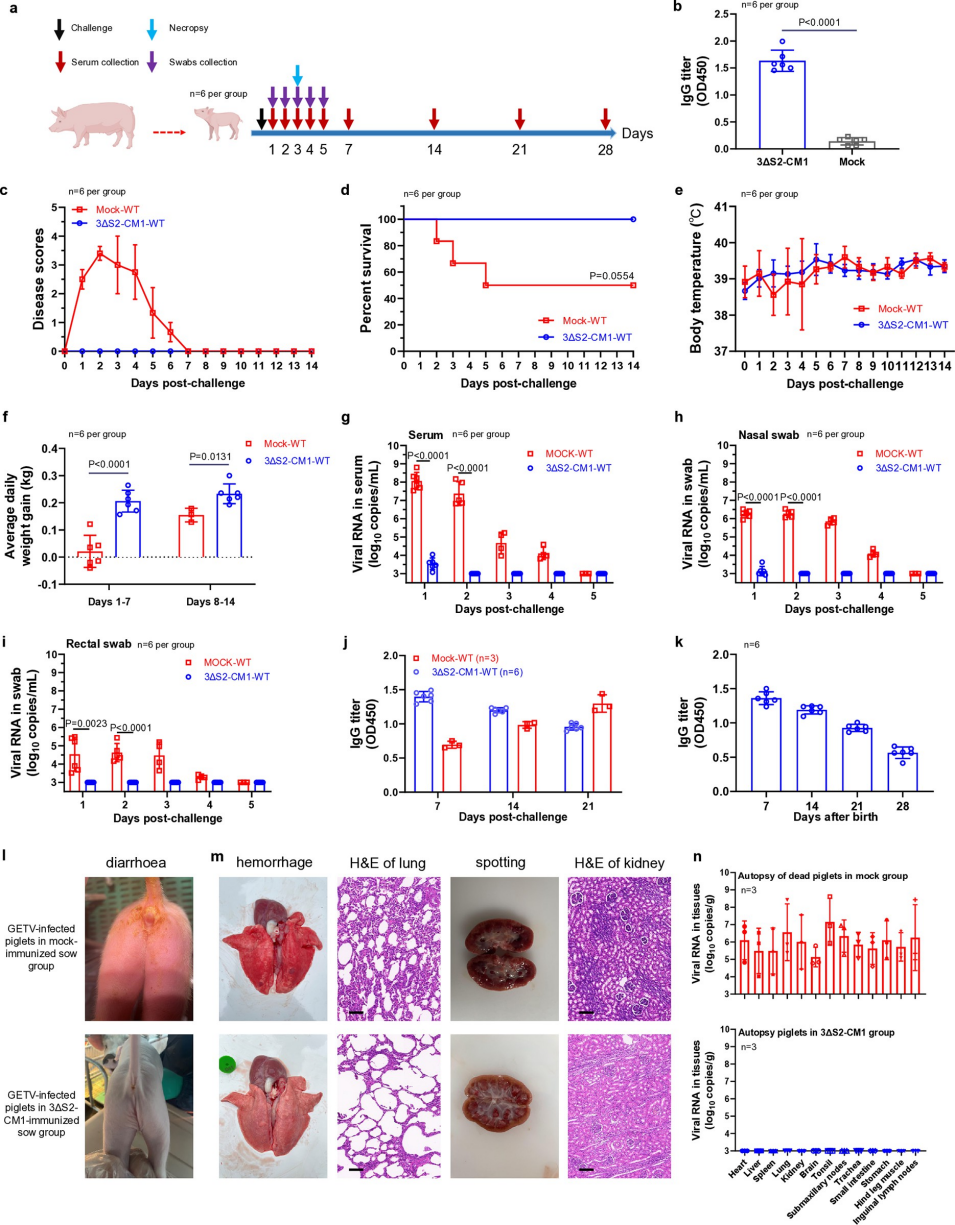
Passive immunization with GETV-3ΔS2-CM1 protects newborn piglets against WT GETV challenge. **(a)** Schematic description of the experiment. Note that the sows are the same animals shown in Fig 5a. Piglets (n = 6 per group) from GETV-3ΔS2-CM1-immunized and mock-immunized sows were fed milk from their mothers for 1 day, after which they were challenged with 5 × 10^7^ TCID_50_ of WT GETV. Body weights and disease scores were monitored daily, and samples were collected at the indicated time points. **(b)** GETV-specific IgG titres in the serum of 1-day-old piglets fed colostrum from immunized and mock-immunized sows were measured using ELISA. **(c)** Disease scores, **(d)** survival, **(e)** body temperatures, and **(f)** average daily weight gains measured for 14 days after challenge. Viral RNA copy numbers in the serum samples **(g)**, nasal swabs **(h)**, and rectal swabs **(i)** at Days 1–5 after challenge were measured using RT-qPCR. **(j)** GETV-specific IgG titres in serum samples from animals from the immunized sow group and surviving animals from the mock immunized sow group at 7, 14, and 21 days after challenge were measured using ELISA. **(k)** GETV-specific IgG titres in serum samples of piglets (n = 6) from the GETV-3ΔS2-CM1-immunized sow group at 7, 14, 21, and 28 days after birth were measured using ELISA. These animals were not challenged with WT GETV. **(l, m)** Representative images illustrating **(l)** the diarrhoea status and **(m)** pathological changes in the lungs and kidneys (right panels show H&E staining, scale bars 100 μm) of the GETV-infected piglets in mock-immunized sow group. Images of animals from the GETV-infected piglets in immunized sow group are provided for comparison. **(n)** Viral RNA copy numbers in different organs of piglets from the mock-immunized sow group that died from WT GETV infection (n = 3). The data from the analysis of the organs of piglets from the immunized sow group that were sacrificed on Day 3 after challenge (n = 3) are shown for comparison. For panels **b**, and **f-i** statistical analysis was performed using Student’s t test. P-values are shown. For panel **d** statistical analysis was performed using the log-rank test. Due to limited number of animals in this experiment the difference did not reach statistical significance (P = 0.0554). *Panel a in this figure was created with BioRender*.

Next, we analysed whether antibodies acquired from the colostrum/milk of immunized sows can protect piglets against challenge with a high dose of WT GETV (Fig 6a). After 1 day of maternal milk feeding, the piglets from immunized animals had high GETV-specific IgG titres in their serum (Fig 6b). After challenge, all piglets in the mock-immunized sow group developed disease; at the same time, no disease signs were observed in the piglets in the immunized sow group (Fig 6c). No animals in the immunized sow group died, while in the mock-immunized sow group, 50% of the animals succumbed to the infection by Day 5 after the challenge (Fig 6d). Some piglets from the mock-immunized sow group also had somewhat reduced body temperatures during the first 7 days after challenge (Fig 6e); this was mainly caused by severe diarrhoea in these animals. Animals from the mock-immunized sow group also displayed reduced weight gain, especially during the symptomatic phase of infection (Days 1–7) (Fig 6f). The levels of GETV RNA in the serum samples, nasal swabs, and rectal swabs of piglets from the immunized sow group were very close (or below) to the detection limit of the RT-qPCR assay. In contrast, high levels of viral RNA were detected in the serum samples of the piglets in the mock-immunized sow group, and viral RNA was also detected in the nasal and rectal swabs of these animals (Fig 6g–6i). In the sera of piglets from the immunized sow group, GETV-specific IgG levels gradually decreased from Day 7 to Day 21 after challenge, indicating a lack of anamnestic response. In contrast, a gradual increase in the titre of GETV-specific antibodies was observed in surviving piglets in the mock-immunized sow group (Fig 6j). In addition, monitoring of maternal antibody levels revealed that the piglets could maintain anti-GETV antibodies for at least 1 month after birth while feeding on maternal milk, although over this period of time, their anti-GETV IgG titres decreased considerably (Fig 6k). The animals from the mock-immunized sow group experienced diarrhoea (Fig 6l) and lung and kidney pathology (Fig 6m). Upon autopsy, GETV RNA copies were detected in all analysed organs, including the heart, liver, lung and intestines, of these animals but not in the organs of piglets from GETV-3ΔS2-CM1-immunized sows (Fig 6n). Taken together, our data revealed that maternal antibodies completely protected piglets from challenge with a high dose of WT GETV.

### Sera from GETV-3ΔS2-CM1-immunized sows protects neonatal mice from WT GETV challenge

One-day-old mice that received intraperitoneally serum collected from three different GETV-3ΔS2-CM1-immunized sows were challenged with a lethal dose of WT GETV at the following day. Mock-immunized mice died on post infection day 5 or earlier. In contrast, all passively immunized animals were completely protected (S7a Fig) and gained weight during the 12-day monitoring period (S7b Fig). Another group of mice received the same treatment and was used for detection of the viral load in different organs. At Day 2 post-challenge, high viral titres were detected in the lung, spleen and muscle tissues of mice in the control group, while no infectious virus was detected in the tissues of passively immunized mice (S7c–S7e Fig). Thus, antibodies from the serum of GETV-3ΔS2-CM1-immunized sows efficiently protect mice against WT GETV infection.

### Immunization with GETV-3ΔS2-CM1 protects mice against infection with other arthritogenic alphaviruses

**Fig 7:**
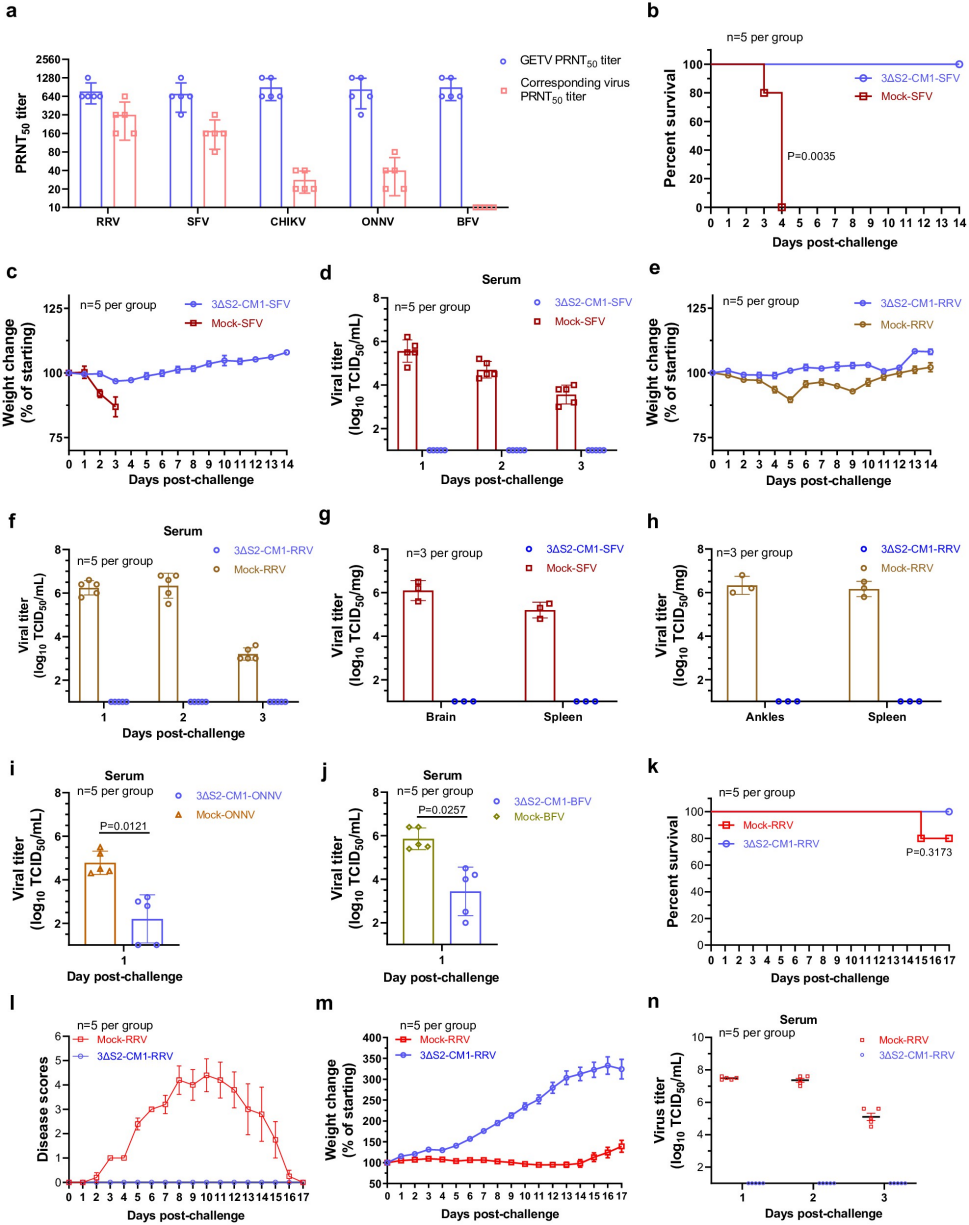
Immunization with GETV-3ΔS2-CM1 protects mice from infection with other arthritogenic alphaviruses. Three-week-old ICR mice (n = 25) were immunized using 1 × 10^5^ TCID_50_ of GETV-3ΔS2-CM1, mice in the control group (n = 20) received PBS. On Day 14 postimmunization, sera from immunized animals were collected, and neutralization titres against GETV (all 25 mice) as well as these against RRV, SFV, CHIKV, ONNV, and BFV (5 mice per virus) were measured using the PRNT assay (**a**). Two days later, the mice were subcutaneously challenged as follows: mice for which neutralization titres against SFV were determined received 1 × 10^4^ TCID_50_ of SFV6, mice for which neutralization titres against RRV were determined received 1 × 10^6^ TCID_50_ of RRV T48, mice for which neutralization titres against ONNV were determined received 1 × 10^6^ TCID_50_ of ONNV, and mice for which neutralization titres against BFV were determined received 1 × 10^6^ TCID_50_ of BFV. For each challenge, control (mock-immunized) mice (n = 5 per group) received the same dose of virus. Survival **(b)**, body weight **(c)**, and viral titres in serum on Day 1, Day 2 and Day 3 **(d)** after challenge with SFV. Body weight **(e)** and viral titres in serum on Day 1, Day 2 and Day 3 **(f)** after challenge with RRV. **(g, h)** Mice were immunized with GETV-3ΔS2-CM1 or mock-immunized as described for (a) and challenged with SFV6 (n = 3 per group) or RRV T48 (n = 3 per group). Viral titres in tissues of SFV **(g)** or RRV **(h)** challenged mice were analysed on Day 3 after challenge. **(i)** Viral titres in the serum of ONNV-challenged mice on Day 1 post infection. **(j)** Viral titres in the serum of BFV-challenged mice on Day 1 post infection. (**k-n**) Six-week-old female ICR mice (n = 3 per group) were inoculated with 1 × 10^5^ TCID_50_ of GETV-3ΔS2-CM1 or mock-inoculated with PBS and mated with male SPF mice 28 days later. The newborn mice were fed by their mothers from birth until the age of 17 days, at which time they were subcutaneously challenged with 1x10^4^ TCID_50_ of RRV T48 (n = 5 in both the immunized and mock-immunized groups). Survival (**k**), disease scores assessed by measuring hind limb condition (**l**), and body weight changes (**m**) were monitored for 17 days. Viral titres in serum samples were analysed at Days 1, 2 and 3 post-challenge (**n**). For panels **i-j** statistical analysis was performed using Student’s t test. For panels **b** and **k** statistical analysis was performed using the log-rank test. P-values are shown.

To determine whether immunization with GETV-3ΔS2-CM1 offers protection against other arthritogenic alphaviruses, 3-week-old mice were subcutaneously inoculated with 1 × 10^5^ TCID_50_ of GETV-3ΔS2-CM1 or mock-immunized with PBS. As expected, the sera collected at 14 days postimmunization efficiently neutralized GETV (PRNT_50_ titre from 320 to 1280; Fig 7a). In addition, efficient neutralization was also observed against RRV and SFV (mean PRNT_50_ titres of 320 and 176). ONNV and CHIKV were neutralized less efficiently (mean PRNT_50_ titres of 40 and 28) and no neutralizing activity against BFV was detected (Fig 7a). Then, the mice were challenged with SFV6, RRV T48, ONNV, or BFV (biosafety level 3 virus CHIKV was omitted from this analysis). It was found that vaccination with GETV-3ΔS2-CM1 conferred complete protection against highly virulent SFV6: all of the mice in the immunized group survived, while all of the mock-immunized mice lost weight and succumbed to the infection by Day 4 post-challenge (Fig 7b and 7c). Furthermore, the GETV-3ΔS2-CM1-immunized mice did not show detectable viremia (Fig 7d). Similar protection was also observed against the virulent T48 strain of RRV. For the first 5 days after challenge, mock-immunized mice lost weight; this was not observed for mice from the immunized group (Fig 7e) and no infectious virus was detected in the sera of these mice (Fig 7f). Similarly, no infectious virus was detected in the organs of GETV-3ΔS2-CM1-immunized mice upon challenge with SFV6 or RRV T48. In contrast, high titres of both of these viruses were detected in the tissues of mock-immunized mice (Fig 7g and 7h). In the mouse models, ONNV and BFV cause only mild signs of disease; therefore, only viral titres in the sera of GETV-3ΔS2-CM1-immunized and mock-immunized mice were compared. In both cases, a significant reduction in viral titres in immunized animals was observed (Fig 7i and 7j). Thus, despite the inability to induce detectable levels of neutralizing antibodies (Fig 7a), GETV-3ΔS2-CM1 immunization also offers protection against BFV.

As immunization with GETV-3ΔS2-CM1 results in passive protection against WT GETV (Fig 3), we used an RRV model to analyse whether this protection also extends to heterologous alphavirus. Six-week-old female mice were immunized with 1 × 10^5^ TCID_50_ of GETV-3ΔS2-CM1 and mated. Pups were fed by their mothers for 17 days, after which they were subcutaneously challenged with 1 × 10^4^ TCID_50_ of RRV T48. No mortality or disease signs were observed in the passively immunized group; the animals gained weight normally. In contrast, all animals in the mock-immunized group developed disease signs following RRV infection, exhibited no weight gain during the first 2 weeks after the challenge and one animal succumbed to the infection (Fig 7k–7m). Consistently, high viremia was observed in mock-immunized but not in GETV-3ΔS2-CM1 immunized group (Fig 7n). Thus, maternal immunization with GETV-3ΔS2-CM1 provided comprehensive protection for their progeny against RRV infection.

### Mutations similar to those present in GETV-3ΔS2-CM1 can be used to attenuate RRV

The sequence diversity complicates copying a design exploited for one alphavirus to another. Here, we took advantage of the fact that the N-terminal region of the alphavirus capsid protein contains multiple positively charged amino acid residues while the HVDs of nsP3 contain multiple proline-rich motifs (S8 Fig) and constructed two mutant variants of RRV, RRV-3ΔS2 and RRV-3ΔS2-CM1 (S9a Fig). In multistep replication analysis in Vero cells, the mutant viruses reached final titres similar to those of the parental RRV T48 strain. However, at early time points, their titres were reduced (S9b Fig), and both mutants produced small plaques on BHK-21 and Vero cells (S9c Fig). Compared with RRV T48, both mutant viruses were attenuated in 17-day-old ICR mice, disease signs were mild, and viral titres in the serum were reduced. While the mutant viruses were generally similar to each other, RRV-3ΔS2-CM1 induced greater lymphocyte infiltration in the hind limbs than RRV-3ΔS2 (S9d–S9h Fig). It should also be noted that unlike in the case of GETV-3ΔS2-CM1, RRV-3ΔS2-CM1 caused detectable disease signs (S9e Fig), either because of the greater initial pathogenicity of RRV T48 in mice or because the sequences affected by the introduced mutations had different impacts on RRV than they had on GETV.

### The GETV-3ΔS2-CM1 backbone can be used for the construction of avirulent chimeric alphaviruses

Chimeric alphaviruses have been generated using SINV or the insect-specific Eilat virus (EILV) as vectors [21,22]. However, these viruses are only distantly related to major human pathogens belonging to the Semliki Forest antigenic complex, such as CHIKV, ONNV, and RRV. Replication of members of the Semliki Forest antigenic complex has a number of specific features, often placing them apart from other alphaviruses [23–26]. Therefore, it is conceivable that using GETV, which belongs to the Semliki Forest antigenic complex, as a vector may have benefits over using SINV or EILV.

**Fig 8:**
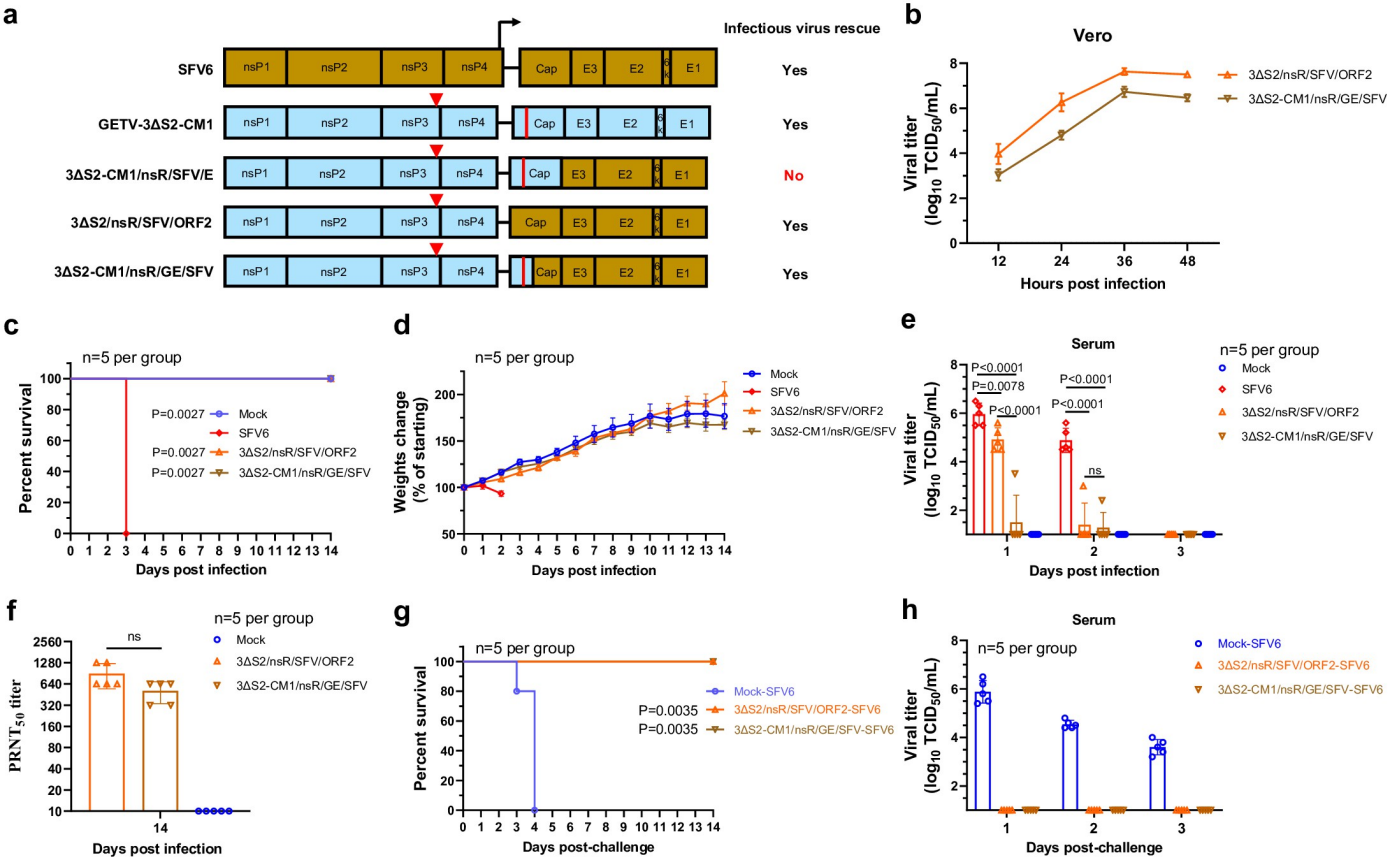
Viable chimaeras based on GETV-3ΔS2-CM1 and SFV are avirulent and protect mice against SFV6 challenge. **(a)** Schematic representation of the genomes of SFV6, GETV-3ΔS2-CM1, 3ΔS2-CM1/nsR/SFV/E, 3ΔS2/nsR/SFV/ORF2, and 3ΔS2-CM1/nsR/GE/SFV. **(b)** Multistep growth curves (MOI = 0.1) of 3ΔS2/nsR/SFV/ORF2 and 3ΔS2-CM1/nsR/GE/SFV in Vero cells. **(c-f)** Three-week-old ICR mice (n = 5 per group) were subcutaneously inoculated with 1 × 10^4^ TCID_50_ of SFV6, 3ΔS2/nsR/SFV/ORF2, 3ΔS2-CM1/nsR/GE/SFV, or with PBS. Survival **(c)** and weight change **(d)** were monitored daily for 14 days. **(e)** Viral titres in sera at Days 1–3 post infection. For SFV6-infected mice, the last time point was not included, as in this experiment all SFV6-infected mice died on Day 3 post infection. **(f)** Neutralization titres against SFV6 in the sera of 3ΔS2/nsR/SFV/ORF2- and 3ΔS2-CM1/nsR/GE/SFV-immunized mice at 14 days post infection. **(g, h)** 3ΔS2/nsR/SFV/ORF2, 3ΔS2-CM1/nsR/GE/SFV, and mock-immunized mice from the previous experiment (n = 5 per group) were challenged with 1 × 10^4^ TCID_50_ of SFV6 at 14 days postimmunization. The survival of the animals was monitored for 14 days after the challenge **(g)**. Serum samples were collected at 1, 2 and 3 days after the challenge, and viral titres were measured **(h)**. For panels **e-f** statistical analysis was performed using Student’s t test. P-values are shown; ns, not significant. For panels **c** and **g** statistical analysis was performed using the log-rank test. P values on panel **c** indicate statistical significance of differences between SFV6 infected animals and mock- or chimaeras infected animals; on panel **g** P-values indicate statistical significance of differences between chimaeras-immunized mice groups and mock-immunized mouse group.

Here, we developed GETV-3ΔS2-CM1-based chimaeras expressing the structural proteins of SFV. SFV was chosen because its strain SFV6 is highly pathogenic for adult mice, making it a sensitive in vivo model [27]. Three chimaeras of GETV-3ΔS2-CM1 and SFV6 were constructed (Fig 8a). Swapping of the entire structural region resulted in a viable virus (3ΔS2/nsR/SFV/ORF2). In contrast, swapping of the glycoprotein (E3-E2-6K-E1) region of GETV-3ΔS2-CM1 with its counterpart from SFV6 resulted in a nonviable construct. To maintain the CM1 mutation, a 3ΔS2-CM1/nsR/GE/SFV chimaera, in which the first 123 amino acid residues of the capsid protein were from GETV-3ΔS2-CM1 and the remaining structural region was from SFV6, was constructed and successfully rescued. In Vero cells, both chimaeras replicated to high titres (Fig 8b) and, in contrast to SFV6, were avirulent in 3-week-old mice (Fig 8c and 8d). These experiments also revealed that 3ΔS2-CM1/nsR/GE/SFV was more attenuated than 3ΔS2/nsR/SFV/ORF2, as it had lower titres in vitro (Fig 8b) and caused much lower viremia than both 3ΔS2/nsR/SFV/ORF2 and SFV6 in mice (Fig 8e). These properties may be attributed to the presence of the CM1 mutation and/or chimeric nature of the capsid protein. Despite the attenuated phenotype, both chimaeras induced seroconversion; the neutralizing antibody titres at 14 days post infection were similar for both viruses (Fig 8f). Furthermore, when the mice were challenged with lethal doses of SFV6, all of the animals immunized with chimeric viruses were completely protected (Fig 8g) and no viremia was observed (Fig 8h). These findings suggest that chimeric viruses similar to 3ΔS2-CM1/nsR/GE/SFV could represent promising vaccine candidates against major human pathogens such as CHIKV, RRV and ONNV.

## Discussion

LAVs are expected to be efficient and also safe to use. The safety of LAVs includes genetic stability, a lack of pathogenicity, and, in the case of arthropod-transmitted pathogens, a lack of vector transmission. These demanding requirements have complicated the development of LAV candidates using traditional methods [5,6]; however, in the case of GETV-3ΔS2-CM1, they were successfully met.

The virulence of alphaviruses is determined by a multitude of viral factors, including nsP3[27]. The C-terminal HVD of nsP3 is highly diverse and primarily mediates the virus-host interactions. Some of these interactions are vital for a virus and cannot be eliminated. For example, the removal of binding sites for Ras-GAP SH3 domain binding proteins (G3BPs), is lethal for CHIKV [28,29]. Elimination of the binding site for FHL1, shown markedly reduce CHIKV virulence [30], cannot be applied for most alphaviruses because, to date, CHIKV and ONNV are the only alphaviruses known to use FHL1 as proviral host factor [31]. Hence, attenuation should be based on altering interactions with common, important, and yet not absolutely required, host components. This suggests, for example, targeting interaction with SH3 domain proteins such as BIN1 and CD2AP, which play redundant roles in the infection of different alphaviruses [19,32]. Mutation 3ΔS1+2 in nsP3 completely eliminated its interaction with BIN1 and CD2AP, while mutations 3ΔS1 or 3ΔS2 did not, indicating that polyproline motifs located in different parts of the HVD of nsP3 of GETV are involved in the interaction with these proteins. The 3ΔS2 mutation reduced the interaction of nsP3 with CD2AP but had little or no effect on interaction with any other host protein previously shown to interact with the nsP3 of alphaviruses that we tested. Thus, the attenuation of GETV-3ΔS2 may be due to weakening of the interaction(s) with one or several known host factors, due to the lack of an interaction with unknown GETV-specific host factor(s) and/or due to a disturbance of other nsP3 function(s).

The N-terminal region of the alphavirus capsid protein is also multifunctional and serves as a virulence determinant. Mutation of positively charged residues located in this region leads to the loss of nuclear localization of the CHIKV capsid protein [17] and alters the nucleolar localization of the capsid protein of EEEV [33]. Changes in the nucleolar localization of the capsid protein were also observed for GETV-CM1 and may be associated with its attenuation. Polybasic sequences located at the N-terminal region of the capsid protein are also needed for interactions with RNA genomes. In addition, they are also required for interaction with interleukin-1 receptor-associated kinase 1 (IRAK1), and the loss of this interaction may also contribute to a decrease in virulence [34]. Thus, specific studies are needed to characterize the molecular mechanisms responsible for GETV-3ΔS2-CM1 attenuation and to reveal how different mutations act synergistically. However, already now data from this and other studies [35] suggest that the synergistic use of two different mutations, each of which likely affects more than one function of the viral protein, can be considered a highly promising approach for the development of LAVs against alphaviruses.

One of the key requirements for studying a virus and developing a LAV is the availability of relevant and sensitive in vivo models. Here, we established a highly sensitive neonatal pig model for lethal GETV infection. Observation that GETV-infected piglets were capable of shedding virus through both nasal secretions and faeces indicates the potential risk of aerosol transmission of GETV, particularly in breeding facilities with suboptimal environmental conditions, and highlights the need for an effective vaccine. When the model was used to characterize the in vivo properties of GETV-3ΔS2-CM1, its replication was found to be short-termed. Interestingly, despite its very low level of replication, GETV-3ΔS2-CM1 was strongly immunogenic. Further studies are needed to reveal the mechanism(s) responsible for this effect; however, it can be speculated that one or both mutations present in the GETV-3ΔS2-CM1 genome reduced the ability of the virus to replicate and/or suppress host antiviral responses and therefore facilitated its detection by the immune system.

Alphaviruses that belong to the same antigenic complex typically induce cross-neutralizing antibodies [30,36]. As expected, immunization with GETV-3ΔS2-CM1 also conferred complete protection against RRV and SFV, which are closely related to GETV (S10a and S10b Fig). Furthermore, sera from immunized mice also neutralized ONNV and CHIKV, important human pathogens. The immunization did not result in detectable levels of antibodies that could neutralize distantly related BFV; however, it did reduce its in vivo replication. Recent studies have demonstrated that the SKT05 antibody, induced by a triple encephalitis VLP vaccine candidate, can effectively protect mice from CHIKV and RRV infections [37]. These findings suggest that there are conserved epitopes in alphaviruses that generate antibodies that are broadly protective, even if some of them do not have significant in vitro neutralizing capability [38]. For alphavirus veterinary vaccines, the ability to protect against multiple viruses has become increasingly important. For example, the recent discovery of the RRV genome in mosquitoes [39] in Yunnan Province, China, suggests that international travel and the horse-racing trade have facilitated the spread of RRV and that it may become endemic in new regions. Single immunization with GETV-3ΔS2-CM1 was sufficient to produce a durable and effective protective effect in animals, eliminating or reducing viremia. From the perspective of One Health approach these properties are crucial because if infected animals exhibit high levels of viremia, they can support the virus transmission cycle, posing a risk of spill-over to people living nearby [40]. This simplified veterinary immunization can considerably reduce the risk as well as the costs associated with human vaccine preparation and administration. Therefore, the findings from this study highlight additional advantages offered by GETV-3ΔS2-CM1 as a promising LAV candidate with broad-spectrum protection against arthritogenic alphaviruses.

LAVs can be highly effective; however, their development using traditional approaches is time-consuming. The rational design of LAVs is hampered by the lack of studies on the virulence factors of most alphaviruses; similarly, the mechanism(s) underlying the attenuation of existing LAVs or LAV candidates are often poorly understood. Finally, as also highlighted by our data, even closely related alphaviruses have different properties, making it difficult to apply a design that is successful for one virus to another. Therefore, a universal strategy, akin to swapping prM and E regions in the yellow fever virus vaccine backbone for flaviviruses [41], can considerably simplify the development of LAVs against alphaviruses. In this regard, the GETV-3ΔS2-CM1 backbone can be considered extremely promising because it originates from a virus not (as of now) associated with human disease, is stably and strongly attenuated by mutations located outside of regions encoding virus glycoproteins and is highly immunogenic. It is also closely related to several major human pathogens, making the design of additional chimeras relatively straightforward. In this study, we provided a proof of concept by demonstrating that GETV-3ΔS2-CM1-based chimeric viruses are safe and efficacious. Importantly, similar to GETV-3ΔS2-CM1 itself, these viruses produce very low levels of viremia, which should prevent infection of mosquitoes via blood feeding and subsequent spread of these recombinant viruses.

In conclusion, we developed an attenuated GETV-3ΔS2-CM1 strain and comprehensively investigated its in vivo characteristics using a novel sensitive and highly relevant newborn pig model. Cross-protection and cross-neutralization experiments revealed the potential of this vaccine candidate against multiple arthritogenic alphaviruses. These data warrant further evaluation using additional models and prevalent circulating virus strains. Furthermore, the GETV-3ΔS2-CM1 backbone was found to represent a promising platform for the development of chimeric vaccines against highly pathogenic alphaviruses, including those infecting humans.

## Materials and methods

### Ethics statements and build-up of in vivo experiments

All animal experiments were conducted following the recommendations outlined in the Guidelines for the Care and Use of Laboratory Animals of the Ministry of Science and Technology of the People’s Republic of China. The experimental protocols were approved by the Animal Experiment Ethics Committee of Nanjing Agricultural University (SYXK2021-0086, NJAU.No20211209191, and NJAU.No20220325062). In experiments with mice and pigs, the number of animals subjected to autopsy was minimized. The sows, piglets, and mice were randomly assigned to different groups. Unless explicitly stated otherwise both male and female animals were used in the experiments. The researchers were unaware of the allocations or outcome assessments during the experiment. All experiments with biosafety level 3 viruses were conducted in the ABSL3 facility of University of Tartu.

### Cells and viruses

BHK-21 (ATCC CCL-10), Vero (ATCC CCL-81), NIH-3T3 (ATCC CRL-1658), HEK 293T (ATCC CRL-3216), and HeLa (ATCC CCL-2) cells were maintained in Dulbecco’s modified Eagle’s medium (DMEM, Gibco) supplemented with 10% foetal bovine serum (FBS, Biological Industries), 100 U/mL penicillin, and 100 μg/mL streptomycin at 37°C in a humidified 5% CO_2_ atmosphere. All of the cell lines tested negative for mycoplasma.

The GETV strains GETV-GX and GETV-FJ were isolated and stored in our laboratory [13]. The wild-type (WT) GETV (strain GETV-HN) [13], SFV (strain SFV6) [42], RRV (strain T48) [43], ONNV (Chad strain) [44], CHIKV (strain LR2006 OPY1) [45], and BFV (strain 2193) [46] were rescued from the corresponding icDNA clones. Briefly, GETV and SFV were rescued by transfection of BHK-21 cells with the corresponding icDNA plasmids. The icDNA plasmids of RRV, ONNV, CHIKV and BFV were first linearized; then 500 ng of DNA was transcribed in vitro using an SP6 mMESSAGE mMACHINE Kit (Ambion) to obtain capped RNA transcripts. icDNA plasmids (2 μg) or 10 μL of RNA transcription mixtures were used to transfect BHK-21 cells using Lipofectamine 2000 transfection reagent and the manufacturer’s (Thermo Fisher Scientific) protocol. Rescued viruses were propagated once in BHK-21 (GETV, RRV, CHIKV and SFV) or Vero cells (ONNV and BFV); the obtained P1 stocks were titered as described below and used for all experiments unless explicitly stated otherwise.

### Antibodies

The following antibodies were used: goat anti-mouse IgG antibody conjugated with horseradish peroxidase (HRP) (Solarbio, 1:10,000 for enzyme-linked immunosorbent assay (ELISA) or western blot), HRP-conjugated goat anti-mouse IgG1 (Biodragon, 100 μL/well for ELISA), HRP-conjugated goat anti-mouse IgG2a (Biodragon, 100 μL/well for ELISA), HRP-conjugated goat anti-mouse IgG2b (Biodragon, 100 μL/well for ELISA), HRP-conjugated goat anti-mouse IgG3 antibodies (Biodragon, 100 μL/well for ELISA), rabbit anti-pig IgG antibody conjugated with HRP (Solarbio, 1:10,000 for ELISA), goat anti-mouse IgG conjugated with FITC (Solarbio, 1:1,000 for IFA), goat anti-mouse IgG conjugated with CoraLite594 (Proteintech, 1:1,000 for IFA), mouse anti-FLAG monoclonal antibody (Sigma, 1:3,000 for western blot), rabbit anti-EGFP polyclonal antibody (Abclonal, 1:2,000 for western blot), and mouse anti-nucleolin antibody (WanleiBio, 1:1,000 for IFA). A GETV E1-3H2 mouse monoclonal antibody (mAb; 1:1,000 for IFA) was made and characterized in house.

### Construction and rescue of GETV mutants, RRV mutants and chimeric viruses

Mutations were introduced into pSM-GETV-HN^FL^, a previously described icDNA clone of GETV-HN [13]. To introduce mutations into nsP3 of GETV, regions corresponding to amino acid residues 358 to 423 (pSM-GETV-3ΔS1), 418 to 453 (pSM-GETV-3ΔS2), or 358 to 453 (pSM-GETV-3ΔS1+2) were replaced with a sequence encoding a short peptide linker (sequence AYRAAG) [16] (S1a Fig). Two mutations were introduced into the structural region: substitution of residues ^69^KPKK^72^ of the capsid protein to ^69^AAAA^72^ (pSM-GETV-CM1) or deletion of the region encoding the 6K protein (pSM-GETV-Δ6K). In the icDNA plasmid designated pSM-GETV-3ΔS2-CM1, the second mutation in nsP3 was combined with substitutions in the capsid protein.

To obtain pRR64-3ΔS2, the region of pRR64 (icDNA clone of RRV T48) corresponding to amino acid residues 428 to 460 of nsP3 was replaced with a sequence encoding an AYRAAG peptide. In pRR64-3ΔS2-CM1, the deletion was combined with the substitution of residues ^70^QRKK^73^ of the capsid protein with ^70^AAAA^73^. In the plasmid pSM-GETV-3ΔS2/nsR/SFV/ORF2, the ORF2 region of GETV-3ΔS2 was replaced with that of SFV6. In the plasmid pSM-GETV-3ΔS2-CM1/nsR/GE/SFV, the region of the structural genes was derived from SFV6, except for the fragment encoding the 123 N-terminal amino acid residues of the capsid protein from GETV-CM1. In the plasmid pSM-GETV-3ΔS2-CM1/nsR/SFV/E, the capsid protein region was from GETV-CM1, and the rest of the structural region, encoding E3-E2-6K-E1, was from SFV6. All plasmids were constructed using PCR-based mutagenesis and restriction enzyme digestion-based cloning. The plasmid sequences were verified by sequencing and are presented in S1 Table.

To rescue mutant viruses, the icDNA plasmids (2 μg) pSM-GETV-3ΔS1, pSM-GETV-3ΔS2, pSM-GETV-3ΔS1+2, pSM-GETV-CM1, pSM-GETV-Δ6K, pSM-GETV-3ΔS2-CM1, or 10 μL of RNA transcription mixtures containing capped RNA transcripts of pRR64-3ΔS2 and pRR64-3ΔS2-CM1 were used to transfect BHK-21 cells. The icDNA plasmids (2 μg) pSM-GETV-3ΔS2/nsR/SFV/ORF2, pSM-GETV-3ΔS2-CM1/nsR/GE/SFV, and pSM-GETV-3ΔS2-CM1/nsR/SFV/E were used to transfect Vero cells. All transfections were performed using Lipofectamine 2000 reagent according to the manufacturer’s protocol. The cell culture supernatants (P0 stocks) were collected upon the development of obvious cytopathic effects (CPEs).

### Virus titration

For the tissue culture infectious dose 50 (TCID_50_) assay, monolayers of BHK-21 or Vero cells grown in 96-well plates were infected using 10-fold serial dilutions of the virus samples prepared in DMEM (100 μL per well). In total, six wells were used for each dilution. The plates were incubated at 37°C for three days, after which wells with CPEs were identified. The TCID_50_ values were calculated using the Reed and Muench formula.

For plaque assays, BHK-21 or Vero cells grown to confluency in 12-well cell culture plates were infected with diluted virus (500 μL per well). After 1 h, the inoculum was removed, and the cells were covered with 1% methylcellulose in DMEM supplemented with 1% FBS, 100 U/mL penicillin, and 100 μg/mL streptomycin. The cells were incubated for 48 or 72 h after which the medium was aspirated, the cells were stained with crystal violet, the plates were extensively washed with water, and the plaques were counted.

### Multistep growth curves and stability assays

Approximately 2 × 10^5^ Vero, BHK-21, or NIH-3T3 cells were seeded per well in 24-well plates. After incubation at 37°C for 24 h, the cells were infected, depending on the virus, at a MOI of 0.01 or 0.1 for 1 h, washed three times with PBS and covered with complete cell culture medium (500 μL per well). At the selected time points, cell culture supernatants were collected and stored at −80°C until titration using the TCID_50_ assay. All experiments were performed in three biological replicates.

To confirm the stability of GETV-3ΔS2-CM1 in vitro, BHK-21 cells were inoculated at a MOI of 0.01 and incubated for 48 h, after which the supernatants were harvested and titrated; the procedure was repeated five times. The supernatant from the fifth passage (BHK P5 stock) was collected, viral RNA was extracted using TRIzol reagent (Vazyme, China), and the presence of the introduced mutations was verified via RT-PCR and sequence analysis of the corresponding regions of the virus genome. To confirm the stability of GETV-3ΔS2-CM1 in vivo, two-day-old suckling mice were subcutaneously inoculated with 3 × 10^5^ TCID_50_ of the virus. Mice were euthanized two days post infection, and spleen and lung tissues were extracted, homogenized and mixed. The obtained material was filtered through a 0.22 μm filter, titered and used for the next passage; this procedure was repeated five times. RNA was extracted from tissue homogenates of the fifth-passage in mice, and the presence of the introduced mutations was verified via RT-PCR and sequence analysis of the corresponding regions of the viral genome. Subsequently, the P5 virus stock from the in vivo experiment was propagated once on BHK-21 cells (mice P5 stock), and its titre was determined. Both P0 and P5 virus stocks from BHK-21 and mice were used to infect two-day-old mice at 3 × 10^5^ TCID_50_ to verify any changes in the pathogenic properties of the viruses.

### Immunoblotting and immunoprecipitation (IP)

The plasmid for the expression of GETV nsP3 with a FLAG-tag at the C-terminus was constructed using the corresponding PCR fragment and pCAGGS expression vector and was designated pCAGGS-nsP3-WT. The plasmids pCAGGS-nsP3-3ΔS1, pCAGGS-nsP3-3ΔS2, and pCAGGS-nsP3-3ΔS1+2, containing the mutations described above, as well as the plasmid pCAGGS-nsP3-ΔHVD, lacking a region corresponding to the HVD of nsP3, were constructed in the same way. Sequences encoding CD2AP, BIN1, CAPZA, CAPZB, PARP1, RPL6, RPL7A, and RPL24 were amplified from mouse cDNAs; the obtained fragments were subsequently inserted into the pEGFP-N1 expression vector. To generate pCap-EGFP and pCM1-EGFP, the regions corresponding to the capsid protein were PCR amplified from pSM-GETV-HN^FL^ and pSM-GETV-3ΔS2-CM1, respectively; the primers were designed such that the cleavage site residue at the C-terminus of the capsid protein was mutated to alanine. The obtained fragments were subsequently inserted into pEGFP-N1 expression vector.

For immunoblotting and IP experiments, equal amounts of plasmids expressing nsP3 or its mutants and plasmids expressing host factors were transfected into HEK 293T cells using Polyethylenimine Linear (PEI) MW40000 reagent (Yeasen Biotechnology); green fluorescence was observed 24 h post transfection to verify the expression of the fusion proteins. Next, the cells were lysed using precooled NP-40 lysis buffer (Beyotime Biotechnology); 30 μL of cell lysates were collected, and 10 μL of 4 × SDS-loading buffer (Solarbio) was added. Proteins were denatured by heating at 95°C for 10 min, separated by SDS–PAGE on 10% gels and transferred to 0.2 μm nitrocellulose membranes (Micropores). The membranes were blocked with a 5% solution of non-fat milk powder in PBST (0.05% Tween 20 in 1 × PBS [pH 7.4]) for 1 h at room temperature and incubated with a mouse anti-FLAG monoclonal antibody or rabbit anti-EGFP polyclonal antibody overnight at 4°C. After washing five times with PBST, the membranes were incubated with an HRP-conjugated goat anti-mouse antibody or goat anti-rabbit antibody for 1 h at room temperature, and the protein bands were visualized using a chemiluminescent HRP detection reagent.

Co-IP of nsP3 and host factors was conducted following previously reported protocols [47]. Briefly, cell lysates obtained as described above were incubated at 4°C for 20 min, after which cell debris was removed by centrifugation at 13,000 × g for 5 min at 4°C. The resulting supernatants were transferred to clean tubes and incubated with 1 μg of anti-FLAG antibody overnight at 4°C, followed by the addition of 20 μL of protein A/G agarose beads (Santa Cruz Biotechnology) for 2 h. Subsequently, the beads were washed four times with NET400 buffer [48] (50 mM Tris [pH 8.0], 400 mM NaCl, 5 mM EDTA, and 1% NP-40) and then mixed with SDS-loading buffer (Solarbio). Proteins were denatured by heating at 95°C for 10 min; the obtained samples were subjected to SDS-PAGE and immunoblot analysis using both anti-EGFP and anti-FLAG tag antibodies as described above.

### Indirect immunofluorescence assays (IFA)

BHK-21 cells grown in 96-well plates were infected with WT GETV or its mutants at a MOI of 0.01. At 12 h post infection (hpi), the media was removed, and the cells were washed with PBST and fixed with 4% paraformaldehyde for 15 min at room temperature. Next, the cells were washed three times with PBST, permeabilized with 0.1% Triton X-100, washed three times with PBST and blocked with a 5% solution of non-fat milk powder in PBST for 1 h. Following three washes with PBST, the cells were incubated with a mouse anti-GETV E1 protein monoclonal antibody (E1-3H2) diluted in PBS for 2 h at room temperature. Following three washes with PBST, the cells were incubated with a FITC-conjugated goat anti-mouse IgG antibody for 1 h at room temperature, washed three times with PBST and stained with 4′,6-diamidino-2-phenylindole (DAPI, 1:1,000; Solarbio) to visualize the nuclei. The samples were analysed using a Nikon confocal microscope.

For detection of proteins via confocal microscopy, equal amounts of plasmids expressing nsP3 or its mutants and plasmids expressing CD2AP or BIN1 were transfected into HeLa cells cultured in glass bottom cell culture dishes (NESTs). The cells were fixed 20 h post transfection and treated as described above; in this experiment, mouse anti-FLAG monoclonal antibody (Sigma) was used as the primary antibody, and goat anti-mouse IgG conjugated with CoraLite594 (Proteintech) was used as the secondary antibody. To monitor the nucleolar localization of capsid proteins, BHK-21 cells were transfected with pCap-EGFP, pCM1-EGFP, or pEGFP-N1. At 24 h post transfection the cells were fixed, stained with an antibody against nucleolin to visualize the nucleolus and with DAPI to visualize the nuclei; the signals and the EGFP autofluorescence were observed by confocal microscopy.

### Mouse experiments

Three-week-old and 6-week-old ICR mice and pregnant ICR mice were purchased from Jiangsu Huachuang Xinuo Medical Technology Co., Ltd., China. Pregnant mice (about 17 days of gestation) were housed individually and gave birth after 4–5 days; the pups were evenly grouped, with a maximum of 10 mice per group. Throughout the experiments, the mice were maintained under ABSL-2 conditions at a temperature between 22 and 25°C under a 12 h/12 h dark/light cycle and provided with standard rodent food and sterile water ad libitum.

To assess the pathogenicity of GETV and its mutant variants, 2-day-old mice (n = 10 per group) were subcutaneously inoculated with 30 μL of PBS containing 3 × 10^5^ TCID_50_ of WT GETV, GETV-CM1, GETV-Δ6K, GETV-3ΔS1, GETV-3ΔS2 or GETV-3ΔS2-CM1; the control group received PBS. Body weight changes and other signs of GETV infection were monitored over a period of 12 days. In another experiment, 2-day-old mice (n = 3 per group) were infected as described above; the animals were euthanized on day 2 post infection, and the viral load in the spleen, lung and muscle tissues was measured using a TCID_50_ assay.

To analyse the immunogenicity of GETV-3ΔS2-CM1, 3-week-old female ICR mice (n = 5 per group) were subcutaneously inoculated with 100 μL of PBS containing 1 × 10^5^ TCID_50_ of WT GETV or GETV-3ΔS2-CM1, and control mice received PBS. Viral titres in serum collected on Day 1 and Day 2 post infection were determined by TCID_50_ assays. At Days 14 and 28 post infection, blood samples were collected via retro-orbital bleeding and used for antibody detection. On Day 30 post infection, the mice were challenged with a subcutaneous injection of 1 × 10^6^ TCID_50_ of WT GETV. Serum samples were collected at 1, 2, and 3 days post-challenge. On Day 3 post-challenge, the mice were euthanized, and a TCID_50_ assay was used to determine the viral loads in the lung, spleen, and muscle.

For the analysis of antigen-specific splenocyte proliferation, 3-week-old female ICR mice (n = 3) were immunized as described above. On Day 14 postimmunization, the animals were euthanized, and their spleens were collected. To monitor the duration of anti-GETV immunity, 3-week-old female ICR mice were immunized (n = 5) or mock-immunized (n = 3) as described above. Serum samples were collected at 2, 4, 6, 9, and 12 months after immunization and used for the measurement of IgG titres and neutralizing antibody levels.

To assess the passive protection resulting from immunization, 6-week-old female ICR mice (n = 3 per group) were subcutaneously immunized with 1 × 10^5^ TCID_50_ of GETV-3ΔS2-CM1 or mock-immunized with PBS. Serum samples were collected on Day 28 postimmunization, and mating was performed on Day 30 postimmunization. To analyse the protection against GETV infection, ten 2-day-old pups per group were subcutaneously inoculated with 30 μL of PBS containing 3 × 10^5^ TCID_50_ of WT GETV. Body weight changes and other signs of GETV infection were monitored over a period of 12 days. To analyse the virus distribution in the organs, 2-day-old pups (n = 10 per group) were subcutaneously inoculated with 30 μL of PBS containing 3 × 10^5^ TCID_50_ of WT GETV. Five mice were euthanized on Day 1, and five mice were euthanized on Day 2 post infection; the amount of viral RNA in different organs was determined using RT-qPCR.

To analyse the passive protection of GETV-3ΔS2-CM1 immunization against challenge with RRV, 6-week-old female ICR mice (n = 5 per group) were immunized or mock-immunized as described above. Serum samples were collected for antibody determination 4 weeks after immunization. Subsequent mating was performed, and the offspring of vaccinated and mock-immunized mothers were continuously fed by their mothers. At the age of 17 days, these mice (n = 5 for the immunized and mock-immunized groups) were subjected to subcutaneous injection of 1 × 10^4^ TCID_50_ RRV T48. Mice were monitored for weight changes and disease progression over a period of 17 days. RRV disease scores were assessed based on strength and hind-leg dysfunction using the following scale: 0, no disease signs; 1, ruffled fur; 2, very mild hindlimb weakness; 3, mild hind limb weakness; 4, moderate hind limb weakness and dragging of hind limbs; 5, severe hind limb weakness/dragging; 6, complete loss of hind limb function; and 7, moribund [49].

One-day-old ICR mice (n = 8 per group) were intraperitoneally injected with 20 μL of D28 serum obtained from three immunized sows or with PBS. On the following day, the mice were subcutaneously challenged with 3 × 10^5^ TCID_50_ of WT GETV; their mortality and weight changes were monitored for a period of 12 days. In another experiment, additional groups of mice were immunized and infected as described above (n = 5 for the PBS group and n = 3 for each serum group). On Day 2 post-challenge, the mice were sacrificed, and the viral loads in the lungs, spleen, and muscles were measured by TCID_50_ assay.

To evaluate the protection of GETV-3ΔS2-CM1-immunized mice against challenge with different GETV strains, 3-week-old ICR mice (n = 9) were immunized with 1 × 10^5^ TCID_50_ of GETV-3ΔS2-CM1, control mice (n = 9) received PBS. Blood samples were collected via retro-orbital bleeding at Day 14 postimmunization. Next, the mice were distributed among three groups (three immunized and three mock-immunized mice per group) and subcutaneously challenged with 1 × 10^6^ TCID_50_ of GETV-HN, GETV-GX or GETV-FJ. Serum samples were collected on Day 1 and Day 2 after challenge and analysed using a TCID_50_ assay. Neutralizing antibody titres in sera collected at 14 days postimmunization and in sera collected at Day 14 post-challenge were measured using PRNT performed using matching GETV strains.

To evaluate the protection of GETV-3ΔS2-CM1-immunized mice against challenge with different alphaviruses, 3-week-old female ICR mice (n = 25) were subcutaneously inoculated with 1 × 10^5^ TCID_50_ of GETV-3ΔS2-CM1; control mice (n = 20) received PBS. Blood samples were collected via retro-orbital bleeding at 14 days postimmunization. Next, the mice were randomly distributed among four groups (five immunized and five mock-immunized mice per group). The sera from five immunized animals was used for detection of neutralizing antibodies against SFV, RRV, ONNV, BFV, and CHIKV. At 16 days postimmunization, mice were challenged subcutaneously infected with respective viruses (except for CHIKV): the first group received 1 × 10^4^ TCID_50_ of SFV6, second group received 1 × 10^6^ TCID_50_ of RRV T48, third group received 1 × 10^6^ TCID_50_ of ONNV, and fourth group received 1 × 10^6^ TCID_50_ of BFV. The animals were weighed daily and monitored for signs of illness throughout the experiment. For SFV- and RRV-challenged mice, serum samples were collected on Days 1, 2 and 3 post-challenge and analysed using a TCID_50_ assay. For ONNV- and BFV-challenged mice, serum samples were collected on Day 1 post-challenge and analysed using a TCID_50_ assay. In another setup, immunized and mock-immunized female mice (n = 3 per group) were challenged with SFV6 and RRV T48 as described above. On Day 3 post-challenge, the mice were euthanized, and viral titres in the spleen and ankle tissues (RRV group) or in the spleen and brain (SFV group) were determined.

To evaluate the attenuation and immunogenicity of the chimeric viruses, 3-week-old female ICR mice (n = 5 per group) were subcutaneously inoculated with 1 × 10^4^ TCID_50_ SFV6, 3ΔS2/nsR/SFV/ORF2, or 3ΔS2-CM1/nsR/GE/SFV, and mock-infected mice received PBS. Mice were monitored for disease signs and weight changes over a period of 14 days. At Days 1, 2 and 3 post infection, serum samples were collected for viremia analysis. Subsequently, at 14 days post infection, serum samples from the surviving animals were collected for antibody testing. Next, the mice were subcutaneously challenged with 1 × 10^4^ TCID_50_ SFV6 and observed for disease signs for 14 days. Viremia was determined at Days 1, 2 and 3 post infection.

To assess the attenuation of RRV mutants, 17-day-old female ICR mice (n = 5 per group) were inoculated subcutaneously with 1 × 10^4^ TCID_50_ of RRV T48, RRV-3ΔS2, or RRV-3ΔS2-CM1 or mock-infected with PBS. Mice were monitored for weight changes and disease progression using the above-described disease scoring system over a period of 17 days. Viremia was determined at Days 1, 2 and 3 post infection. In another setup, mice infected or mock-infected as described above (n = 3 per group) were sacrificed on Day 10 post infection, and the quadriceps femoris muscles were harvested and subjected to haematoxylin and eosin (H&E) staining.

### Pig experiments

Piglets used for comparison of pathogenicity of GETV and GETV-3ΔS2–CM1 (Fig 4) as well as pregnant Yorkshire sows lacking anti-GETV antibodies and that tested negative according to (RT)-PCR for porcine reproductive and respiratory syndrome virus (PRRSV), porcine circovirus (PCV), pseudorabies virus (PRV), classical swine fever virus (CSFV), African swine fever virus (ASFV), and JEV were purchased from a local farm with high biosecurity standards and hygiene. The sows were individually housed for 35 days before parturition. During the infection and immunization experiments, the sows and piglets were housed in standard farrowing crates and provided with gestation-specific food and sterile water.

To assess the pathogenicity of GETV and GETV-3ΔS2-CM1 in piglets, thirteen animals purchased from a local farm were randomly divided into three groups. In the first group, 1-day-old piglets (n = 5) were infected with 5 × 10^7^ TCID_50_ of WT GETV simultaneously via three routes: subcutaneous (2 × 10^7^ TCID_50_), intravenous (2 × 10^7^ TCID_50_) and intramuscular (1 × 10^7^ TCID_50_) inoculation. The second group of 1-day-old piglets (n = 5) were infected in the same way except that GETV-3ΔS2-CM1 was used. In the third group, 1-day-old piglets (n = 3) were mock-infected with saline solution via the schema described above. Piglets were monitored daily with measurement of rectal temperature and for the presence or absence of diarrhoea, hind limb paralysis and depression. Diarrhoea was assessed by scoring the stool consistency as follows: 0, normal solid stool; 1, pasty stool; 2, semisolid/liquid stool; and 3, stool without solid components. In addition, a score of 1 was given to piglets with signs of mental depression or hind limb paralysis. The overall disease score was based on the sum of the stool score and the mental status/paralysis score, with a maximum value of 4. Piglets without any signs of disease were scored 0.

Serum samples were collected at Days 1–5 after infection and used for detection of viral RNA. Serum samples from surviving animals were collected at 14 days post infection and used for the detection of GETV-specific IgG antibodies. Upon reaching the clinical endpoint, the animals were euthanized and immediately autopsied. In an additional experiment, a group of 1-day-old piglets (n = 7) purchased from a local farm was infected with GETV-3ΔS2-CM1 as described above. Two animals were euthanized on Day 1, two animals were euthanized on Day 2, and three animals were euthanized on Day 3; autopsy was performed immediately, and the viral loads in the organs were determined.

To assess the passive protection resulting from immunization with GETV-3ΔS2-CM1, pregnant sows with an expected parturition in approximately 35 days were immunized by intramuscular injection of 1 × 10^5^ TCID_50_ of GETV-3ΔS2-CM1 in the neck region (n = 3) or mock-immunized using saline (n = 2). Nasal swabs, rectal swabs and serum samples to be used for virus detection were collected on Days 1–5 postimmunization; serum samples for anti-GETV antibody detection were collected on postimmunization Days 7, 14, 21, 28 and 35. Cord blood samples were collected at parturition, and maternal milk samples were collected on Days 1–10 after parturition. After birth, the piglets were nursed by their mothers. Serum samples were collected from 1-day-old piglets, after which the animals (n = 6 for the immunized group and n = 6 for the mock-immunized group) were challenged with WT GETV as described above. Changes in body temperature, body weight, and disease scores were continuously recorded for 14 days. Serum samples, nasal swabs and rectal swabs were collected from the surviving piglets for 1–5 days after challenge and used to analyse the presence of viral RNA. Three piglets from mock-immunized sows that reached clinical endpoint were euthanized, and tissue samples were collected. To obtain comparable samples from the GETV-3ΔS2-CM1 group, three more piglets from immunized sows were challenged with WT GETV and euthanized on Day 3 post-challenge, after which tissue samples were collected.

To evaluate the persistence of maternal antibodies in piglets, five piglets born to immunized sows were kept unchallenged. Serum samples were collected at 7, 14, 21 and 28 days of age and used for the analysis of GETV-specific antibodies.

### Enzyme-linked immunosorbent assay (ELISA)

The sera collected at the indicated time points were used to assess anti-GETV antibody titres following a previously reported protocol [50]. Briefly, ELISA plates were coated with human cell-derived recombinant p62-E1 protein from GETV. One hundred microlitres of protein solution (0.5 μg/mL) was added to each well, and coating was performed overnight at 4°C. The coated plates were washed five times with PBST and blocked with a 5% solution of non-fat milk powder in PBST for 1 h; this was followed by five washes with PBST. The collected serum samples were diluted 1:100 with PBST and added to each well (100 μL/well). The plates were incubated at 37°C for 1 h, washed five times with PBST, and HRP-conjugated rabbit anti-pig IgG, HRP-conjugated goat anti-mouse IgG, HRP-conjugated goat anti-mouse IgG1, HRP-conjugated goat anti-mouse IgG2a, HRP-conjugated goat anti-mouse IgG2b, or HRP-conjugated goat anti-mouse IgG3 antibodies were added, followed by incubation at 37°C for 45 min. The plates were washed five times with PBST, and the chromogenic substrate (100 μL/well) was added. After incubation at 37°C for 15 min, the reaction was terminated by the addition of stop solution, and the absorbance at 450 nm (OD450) was measured using an ELISA reader. The cut-off value of this assay was OD450 = 0.3565.

### Plaque reduction neutralization tests (PRNTs)

Two-fold serial dilutions of sera were prepared using DMEM containing 2% FBS and 1% penicillin/streptomycin; the starting dilution was 1:20 (mouse sera) or 1:10 (pig sera), and the maximum dilution was 1:2,560 for both sera. The diluted sera (100 μL) were incubated with an equal volume of corresponding GETV stock containing 1 × 10^3^ TCID_50_/mL at 37°C for 1 h; in the blank control, GETV was incubated with DMEM containing 2% FBS and antibiotics. After incubation, the samples were used to infect BHK-21 cells grown in 24-well plates; infected cells were covered with medium containing 1% methylcellulose. After 48 h, the medium was aspirated, and the cell monolayer was stained with crystal violet, followed by extensive washing with water. The PRNT assays using SFV, RRV, CHIKV were performed at the same way except 72 h infection time was used for SFV and RRV. CHIKV experiments were performed in the BSL3 facility of the University of Tartu. The PRNT assays using ONNV and BFV were performed using Vero cells and 72 h infection time. The titres of neutralizing antibodies (PRNT_50_ values) in serum samples were calculated as the highest dilution at which a >50% reduction in plaque count (compared to the blank control) was observed.

### Spleen lymphocyte proliferation assay

Splenocytes from mice were aseptically isolated using a 70-mesh cell sieve, treated twice with erythrocyte lysis buffer, and diluted to 2.5 × 10^6^ cells/mL in RPMI 1640 medium. Cells were plated into 96-well plates at 100 μL of cell suspension per well. Three pair of wells were used for cells from each animal; in addition, three wells with 100 μL of RPMI 1640 medium in the absence of cells were used as a blank control. Subsequently, 10 μL of a solution of purified recombinant GETV p62-E1 protein (stimulant; 10 μg/mL) or RPMI medium (control) was added to the wells, after which the plates were incubated for 36 h at 37°C. Next, 10 μL of CCK-8 reagent (Yeasen Biotechnology, China) was added to each well, the plates were incubated for 4 h at 37°C, and the OD450 values were detected using a multifunctional plate reader. The splenocyte stimulation index was calculated as follows: SI = (OD stimulant—OD 1640)/(OD control—OD 1640) where OD 1640 represents an OD450 for the blank control.

### RNA extraction and RT-qPCR

The euthanized animals were dissected, and heart, liver, spleen, lungs, kidneys, and other tissues were collected and weighed. A volume of PBS corresponding to the mass of the sample was added, the tissues were homogenized and centrifuged at 13,000 × g for 5 min. In the case of swabs, 1 mL of PBS was added to the samples, and the probes were vortexed and centrifuged at 13,000 × g for 3 min. Then, 200 μL of the obtained supernatant was mixed with 800 μL of TRIzol reagent. For the serum samples, the homogenization steps were omitted, and 200 μL of serum was mixed with 800 μL of TRIzol reagent. The same approach was used for supernatants collected from infected NIH-3T3 cells. To obtain intracellular RNAs from NIH-3T3 cells the infected cells were washed, suspended in PBS after which 500 μL of TRIzol reagent was added. RNA was extracted according to the manufacturer’s instructions.

cDNA synthesis was performed using a Vazyme (Nanjing, China) kit; TaqMan qPCR was performed using primers and probes targeting the nsP4 gene of GETV (nsP4-qPCR-F: 5’-ATGAAGACGAACGCATATACCC-3’; nsP4-qPCR-R: 5’-CATGTTCTCTACTTTCCTCGAC-3’; nsP4-qPCR-Probe: 5’-FAM-CCGACAGAAGCGAATAAG-MGB-3’). Quantification was performed using a standard curve obtained using 10-fold serial dilutions of plasmid containing DNA copy of the corresponding fragment of the nsP4 gene. The reaction was carried out using qPCR Probe Master Mix (Vazyme) and the following reaction parameters: (1) 95°C for 10 min, (2) 95°C for 10 s, 59°C for 10 s, and 72°C for 15 s for 45 cycles. The detection limit of the assay was 10 RNA copies/μL; samples with Cq values greater than 35 were considered negative. Quantification of mRNA of IFN-β was performed as follows. 1 μg of total RNA was used for reverse transcription. Gene expression assessment was done using primers specific for the IFN-β gene (F: 5’-ACAGCCCTCTCCATCAACTATAAG-3’, R: 5’-ATCTTCTCCGTCATCTCCATAGGG-3’); primers specific for mouse GAPDH gene (F: 5’-CCACTCACGGCAAATTCAAC-3’, R: 5’-CTCCACGACATACTCAGCAC-3’) were used to quantify the internal control. The reaction was carried out using SYBR qPCR Master Mix (Vazyme) and the following reaction parameters: (1) 95°C for 10 min, (2) 95°C for 10 s and 60°C for 30 s for 45 cycles, (3) 95°C for 10 s, 65°C for 60 s, and 97°C for 1 s. The data were analysed using the cycle threshold (2^−ΔΔCt^) method [13].

### Histological examination

During autopsy, the lungs and kidneys of euthanized piglets were carefully excised. Representative portions of the lungs and kidneys were collected in 10% buffered formalin for histological analysis. Following collection, the tissues were embedded in paraffin wax, and the paraffin blocks were cut into 4 μm thick sections. Sections were mounted onto glass slides, stained with H&E and examined under a light microscope to assess histological changes or abnormalities present in the tissues.

### Statistical analysis

The data are presented as the mean ± standard deviation (SD) from three or more independent experiments. The data were analysed, and statistical analysis was performed using GraphPad Prism 9 (GraphPad Software, San Diego, CA, USA). The significance of differences between groups was determined by Student’s t test, except for comparisons of survival curves, for which the log-rank test was used. P values < 0.05 were considered to indicate statistical significance.

## Supporting information

S1 Fig Mutations introduced into the GETV genome and validation of potential mechanisms of virus attenuation. **(a)** Mutations introduced into the HVD of nsP3 of GETV. The G3BP binding motifs, which are crucial for the replication of Old World alphaviruses, are annotated in red. Amino acid residues removed by 3ΔS1 are highlighted in yellow, those removed by 3ΔS2 are highlighted in green, and those removed by 3ΔS1+2 are highlighted in blue. Positions of the first and the last residue in the corresponding protein are indicated. **(b-c)** HEK 293T cells were cotransfected with expression plasmids of mouse CD2AP-EGFP **(b)** or mouse BIN1-EGFP **(c)** and expression plasmids of FLAG-tagged GETV nsP3 (WT), nsP3-3ΔS1, nsP3-3ΔS2, nsP3-3ΔS1+2, nsP3-ΔHVD, or an empty vector (Vec). The cells were lysed, and immunoprecipitation was performed using anti-FLAG antibodies. Proteins present in the lysate (Input) and immunoprecipitated (IP) proteins were detected using anti-FLAG and anti-EGFP antibodies; GAPDH (loading control) was detected in the lysates using the corresponding antibody. Representative images from three independent experiments are shown. Relative intensity above the panels **b** and **c** represents quantity of immunoprecipitated CD2AP-EGFP or BIN1-EGFP proteins. Quantification was performed using ImageJ software. For each panel the amount of precipitated EGFP fusion protein was normalized to the amount of precipitated nsP3 and the ratio for proteins precipitated using WT nsP3 was taken as 1. **(d)** HeLa cells were cotransfected with the above-described combinations of expression plasmids or only with mouse CD2AP-EGFP or mouse BIN1-EGFP fusion protein expression plasmids. Cells were fixed at 24 h post transfection and stained with anti-FLAG and CoraLite594-conjugated goat anti-mouse antibodies; fusion proteins were detected via EGFP fluorescence. Images were acquired using Nikon confocal laser scanning microscope. Representative images from three independent experiments are shown. Scale bar 10 μM. **(e)** (Left panel) Expression of the WT capsid protein of GETV fused with EGFP (Cap-EGFP), its counterpart harbouring the 69-KPKK-72 to 69-AAAA-72 mutation (CM1-EGFP) and EGFP in BHK-21 cells transiently transfected with the corresponding expression plasmids. Proteins were detected using an anti-EGFP antibody; GAPDH (loading control) was detected using the corresponding antibody. (Right panel) Subcellular localization of EGFP, Cap-EGFP and CM1-EGFP in transiently transfected BHK-21 cells at 24 h post transfection. The cells were stained with an antibody against nucleolin to visualize the nucleolus and with DAPI to visualize the nuclei. EGFP and fusion proteins were detected using EGFP fluorescence; images were acquired using Nikon confocal laser scanning microscope. Scale bar 20 μM.
(TIF)

S2 Fig The 3ΔS2 mutation do not abolish the interactions of nsP3 of GETV with multiple host factors. HEK 293T cells were cotransfected with the expression plasmids of mouse CAPZA-EGFP, CAPZB-EGFP, PARP1-EGFP, RPL6-EGFP, RPL7A-EGFP, or RPL24-EGFP and the expression plasmids of FLAG-tagged GETV nsP3 (WT) or nsP3-3ΔS2. The cells were lysed, and immunoprecipitation was performed using anti-FLAG antibodies. Proteins present in the lysate (input) and immunoprecipitated (IP) proteins were detected using anti-FLAG and anti-EGFP antibodies; GAPDH (loading control) was detected in the lysates using the corresponding antibody. Representative images from three independent experiments are shown.
(TIF)

S3 Fig GETV-3ΔS2-CM1 is strongly attenuated in NIH-3T3 cells. NIH-3T3 cells were infected with WT GETV and GETV-3ΔS2-CM1 at a MOI of 0.1. (**a**) Multistep growth curves. (**b)** Copy numbers of viral genomic RNAs in the cell lysate and (**c**) in supernatant were measured using RT-qPCR. (**d**) Relative levels of IFN-β mRNA in WT GETV and GETV-3ΔS2-CM1 infected cells were measured using RT-qPCR. Induction of IFN-β mRNA expression (fold change compared to that of GAPDH mRNA) in infected cells was calculated and obtained values were normalized to the fold changes in WT GETV infected cells (taken as 1). All experiments were performed in three biological replicates, error bars indicate standard deviation.
(TIF)

S4 Fig GETV-3ΔS2-CM1 is genetically stable and maintains an avirulent phenotype in mice. Suckling mice (n = 10 per group) were infected with a P0 stock of GETV-3ΔS2-CM1 or with viruses passaged five times in BHK-21 cells (BHK P5) or in suckling mice (Mice P5). The experiment was performed as described in the legend of Fig 1g. Survival **(a)** and body weight changes **(b)** were monitored daily. An additional group of mice (n = 3 per group) was infected with P0 stock, BHK P5 stock, or Mice P5 stock of GETV-3ΔS2-CM1. Mice were sacrificed on Day 2 post infection, and the viral loads in the spleen, lung, and muscle tissues were measured as described in the legend of Fig 1i. For panel **c** statistical analysis was performed using Student’s t test; ns, not significant.
(TIF)

S5 Fig GETV-3ΔS2-CM1 immunization induces dominantly IgG2 subtype. Three-week-old ICR mice (n = 5 per group) were infected with 1 × 10^5^ TCID_50_ of WT GETV or GETV-3ΔS2-CM1. Isotypes of GETV-specific IgG antibodies in the serum at Day 28 post infection with WT GETV (**a**) or GETV-3ΔS2-CM1 (**b**) were measured by ELISA performed using recombinant GETV p62-E1 protein; titres of IgG1, IgG2a, IgG2b and IgG3 are presented as OD450 values. (**c**) The ratio of IgG2a/IgG1 isotypes in sera of WT GETV and GETV-3ΔS2-CM1 infected mice. For panel **c** statistical analysis was performed using Student’s t test.
(TIF)

S6 Fig GETV-3ΔS2-CM1 immunization protects mice from infection with heterologous GETV strains. Three-week-old ICR mice (n = 9 per group) were immunized with 1 × 10^5^ TCID_50_ of GETV-3ΔS2-CM1 or mock-immunized with PBS. Day 14 postimmunization serum samples were collected and the mice (n = 3 per immunized and mock-immunized group) were subcutaneously challenged with 1 × 10^6^ TCID_50_ of GETV-HN (WT GETV), GETV-GX or GETV-FJ. (**a**) Viral titres in the sera of the GETV-3ΔS2-CM1 and mock-immunized mice at Day 1 and 2 post infection were determined using a TCID_50_ assay. (**b**) Neutralizing antibody titres in sera collected at Day14 postimmunization (IM-D14) and in sera collected at Day 14 post-challenge (C-D14) were measured using PRNT assay performed with matching GETV strains. Statistical analysis was performed using Student’s t test; ns, not significant.
(TIF)

S7 Fig Sera from GETV-3ΔS2-CM1-immunized sows protect neonatal mice from WT GETV infection. One-day-old mice (n = 8 per group) were intraperitoneally injected with 20 μL of serum collected from three GETV-3ΔS2-CM1-immunized sows on Day 28 postimmunization, and control animals were injected with PBS. Twenty-four hours later, the mice were subcutaneously challenged with 3 × 10^5^ TCID_50_ of WT GETV. **(a)** Survival and **(b)** body weight changes were recorded daily. **(c-e)** One-day-old mice (n = 5 in the PBS group and n = 3 in each the serum group) were treated as described above, challenged with WT GETV and sacrificed on Day 2 after challenge. Viral loads in the lung **(c)**, spleen **(d)**, and muscle **(e)** were determined. For panel **a** statistical analysis was performed using the log-rank test. P-values are shown.
(TIF)

S8 Fig The HVD of nsP3 in arthritogenic alphaviruses contains multiple proline-rich motifs. The HVDs of nsP3 of alphaviruses have low sequence similarity and cannot be reliably aligned. Instead, they contain short sequence motifs used to interact with host proteins. G3BP binding motifs crucial for the replication of Old World alphaviruses are annotated in red. In addition, the HVDs of nsP3 in GETV, RRV, SFV, CHIKV, ONNV, MAYV, BFV and UNAV contain multiple proline-rich motifs annotated in dark cyan. Residues deleted in GETV-3ΔS2-CM1 and RRV-3ΔS2-CM1 are shown in bold, and the four (GETV) or three (RRV) proline-rich motifs, removed by deletions, are annotated in blue. Positions of the first and the last residue in the corresponding protein are indicated.
(TIF)

S9 Fig RRV-3ΔS2 and RRV-3ΔS2-CM1 are attenuated in vitro and have reduced pathogenicity in mice. **(a)** Schematic representation of the genomes of RRV T48, RRV-3ΔS2, and RRV-3ΔS2-CM1; the mutations are marked as in Fig 1a. **(b)** Multistep growth curves (MOI of 0.1) of RRV T48, RRV-3ΔS2, and RRV-3ΔS2-CM1 in Vero cells. P values indicate statistical significance of differences between titres of RRV T48 and its mutant variants at 12 hpi. **(c)** Plaque morphologies of RRV T48, RRV-3ΔS2, and RRV-3ΔS2-CM1 on BHK-21 and Vero cells. **(d-f)** Seventeen-day-old ICR mice (n = 5 per group) were subcutaneously inoculated with 1 × 10^4^ TCID_50_ of RRV T48, RRV-3ΔS2, RRV-3ΔS2-CM1 or mock-inoculated with PBS. Survival **(d)**, disease score **(e)**, and weight change **(f)** were monitored daily. **(g)** Viral titres in serum samples from RRV T48-, RRV-3ΔS2-, and RRV-3ΔS2-CM1-infected mice (n = 5 per group) at Days 1, 2 and 3 post infection. (**h**) H&E staining of the quadriceps femoris muscle from mice infected with RRV T48, RRV-3ΔS2, or RRV-3ΔS2-CM1 or mock-infected with PBS at 10 days post infection. Representative images are shown, (scale bar 100 μm). For panels **b** and **g** statistical analysis was performed using Student’s t test. For panel **d** statistical analysis was performed using the log-rank test. P-values are shown; ns, not significant.
(TIF)

S10 Fig Comparison of E3-E2-6K-E1 protein sequences of GETV strains and other arthritogenic alphaviruses used in the study. (**a**) Phylogenetic tree constructed using Lasgene7.1 software. Sequence alignment was performed by the Clustal W method. The percentage of amino acid sequence identity between GETV-HN (WT GETV) and other viruses is presented on the right. (**b**) Alignment of E3-E2-6K-E1 protein sequences of GETV-HN, GETV-GX, GETV-FJ, RRV, SFV, CHIKV, ONNV, and BFV. Amino acid residues are numbered according to the residues in the E3-E2-6K-E1 of GETV-HN.
(TIF)

S1 Table The plasmid sequences of icDNA clone in this manuscript. (XLSX)

S1 Data Raw Data that underlies this paper. (XLSX)
