# Supplementary figures and images for: Attenuated Getah virus confers protection against multiple arthritogenic alphaviruses
Source: PLoS Pathog. 2024 Nov 18;20(11):e1012700. doi: 10.1371/journal.ppat.1012700 (PMC11630583; doi:10.1371/journal.ppat.1012700)

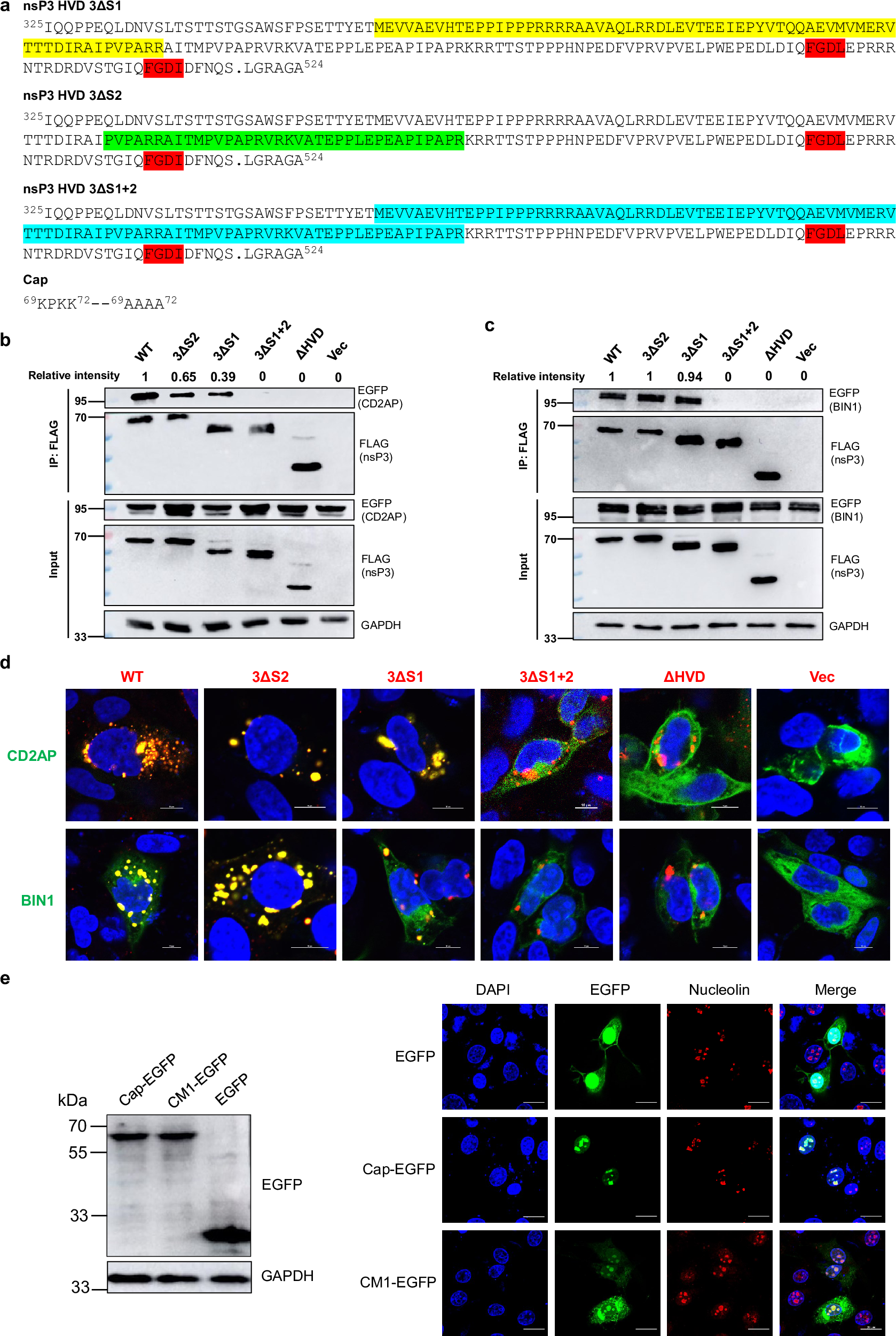

Supplement: S1 Fig — (a) Mutations introduced into the HVD of nsP3 of GETV. The G3BP binding motifs, which are crucial for the replication of Old World alphaviruses, are annotated in red. Amino acid residues removed by 3ΔS1 are highlighted in yellow, those removed by 3ΔS2 are highlighted in green, and those removed by 3ΔS1+2 are highlighted in blue. Positions of the first and the last residue in the corresponding protein are indicated. (b-c) HEK 293T cells were cotransfected with expression plasmids of mouse CD2AP-EGFP (b) or mouse BIN1-EGFP (c) and expression plasmids of FLAG-tagged GETV nsP3 (WT), nsP3-3ΔS1, nsP3-3ΔS2, nsP3-3ΔS1+2, nsP3-ΔHVD, or an empty vector (Vec). The cells were lysed, and immunoprecipitation was performed using anti-FLAG antibodies. Proteins present in the lysate (Input) and immunoprecipitated (IP) proteins were detected using anti-FLAG and anti-EGFP antibodies; GAPDH (loading control) was detected in the lysates using the corresponding antibody. Representative images from three independent experiments are shown. Relative intensity above the panels b and c represents quantity of immunoprecipitated CD2AP-EGFP or BIN1-EGFP proteins. Quantification was performed using ImageJ software. For each panel the amount of precipitated EGFP fusion protein was normalized to the amount of precipitated nsP3 and the ratio for proteins precipitated using WT nsP3 was taken as 1. (d) HeLa cells were cotransfected with the above-described combinations of expression plasmids or only with mouse CD2AP-EGFP or mouse BIN1-EGFP fusion protein expression plasmids. Cells were fixed at 24 h post transfection and stained with anti-FLAG and CoraLite594-conjugated goat anti-mouse antibodies; fusion proteins were detected via EGFP fluorescence. Images were acquired using Nikon confocal laser scanning microscope. Representative images from three independent experiments are shown. Scale bar 10 μM. (e) (Left panel) Expression of the WT capsid protein of GETV fused with EGFP (Cap-EGFP), its counterpa [file ppat.1012700.s001.tif]

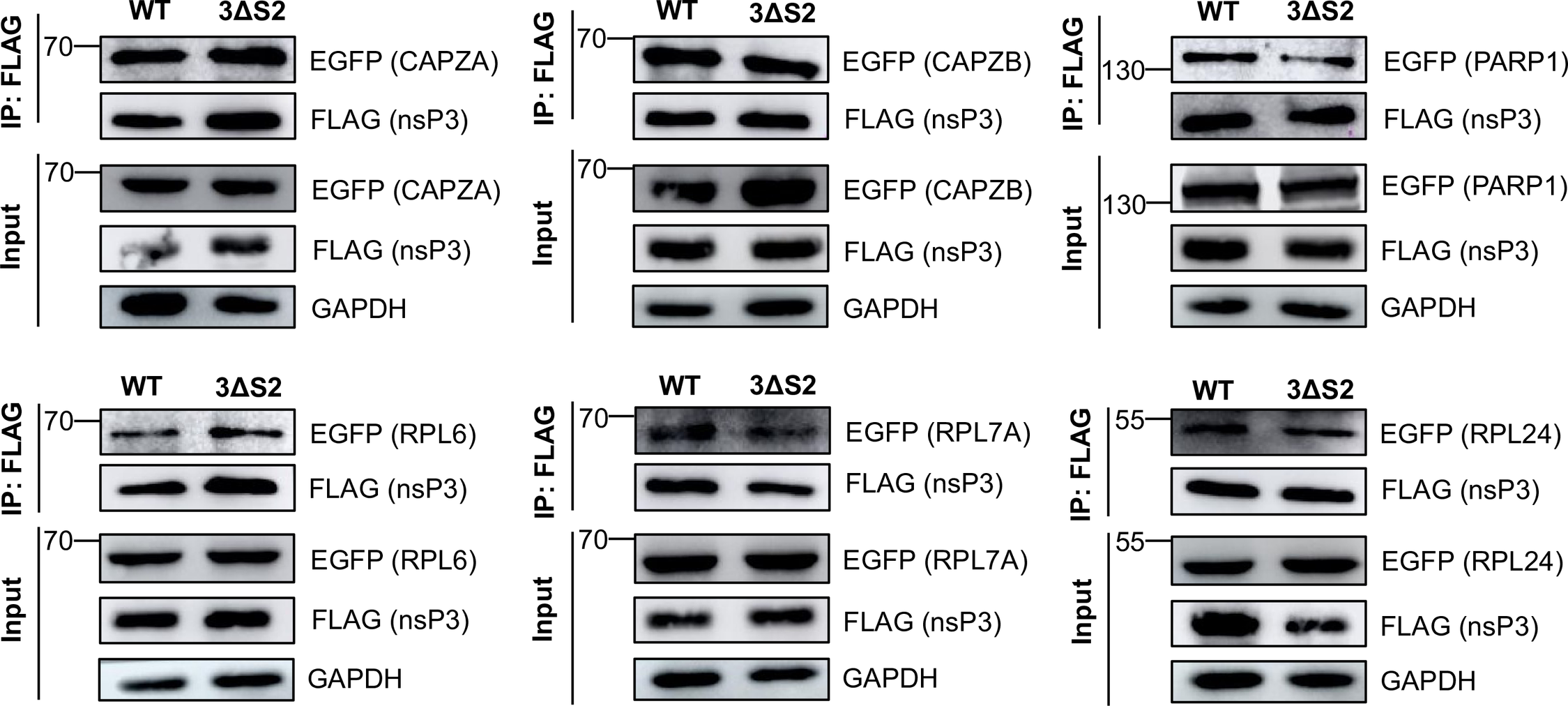

Supplement: S2 Fig — HEK 293T cells were cotransfected with the expression plasmids of mouse CAPZA-EGFP, CAPZB-EGFP, PARP1-EGFP, RPL6-EGFP, RPL7A-EGFP, or RPL24-EGFP and the expression plasmids of FLAG-tagged GETV nsP3 (WT) or nsP3-3ΔS2. The cells were lysed, and immunoprecipitation was performed using anti-FLAG antibodies. Proteins present in the lysate (input) and immunoprecipitated (IP) proteins were detected using anti-FLAG and anti-EGFP antibodies; GAPDH (loading control) was detected in the lysates using the corresponding antibody. Representative images from three independent experiments are shown. (TIF) [file ppat.1012700.s002.tif]

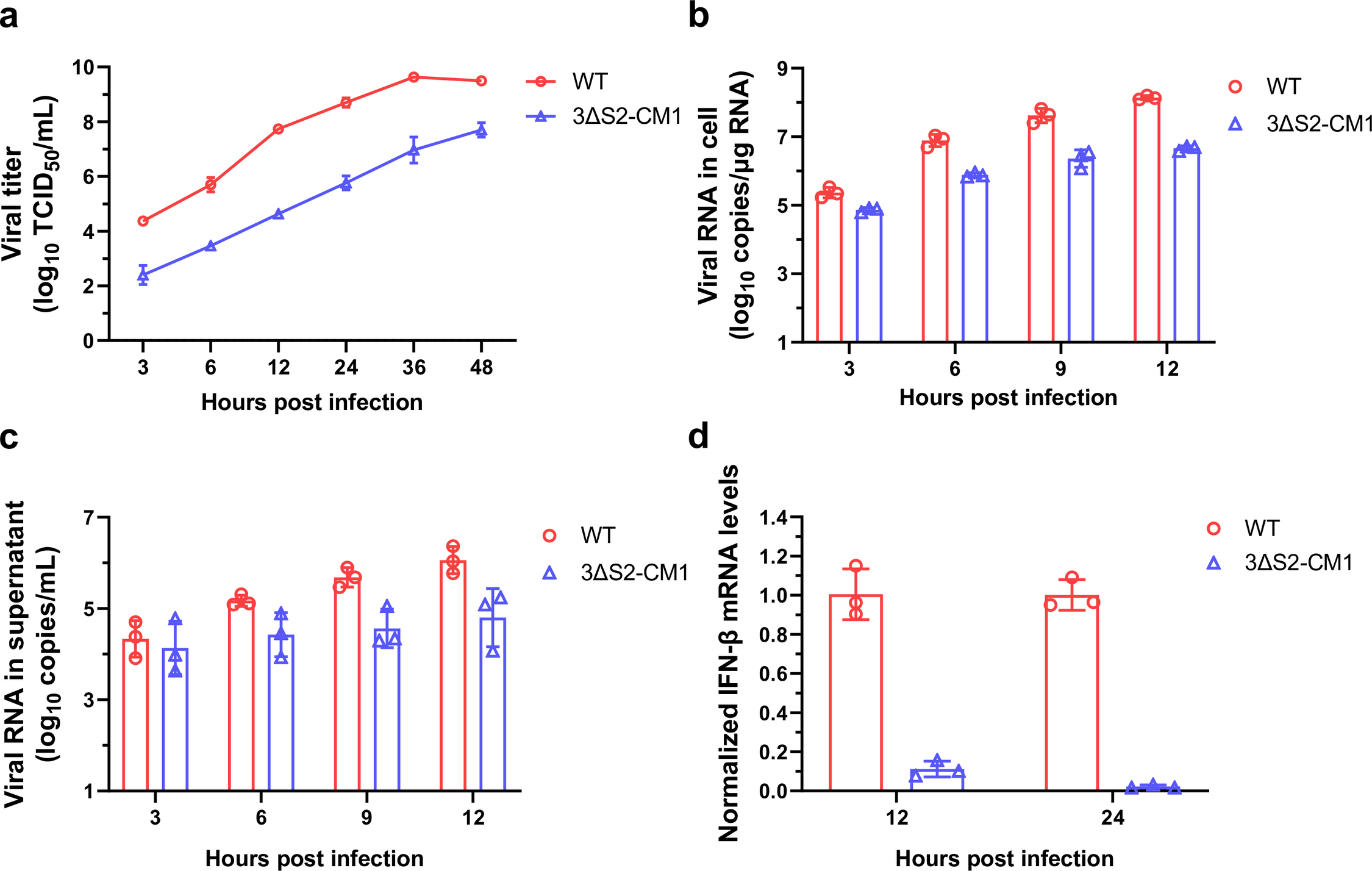

Supplement: S3 Fig — NIH-3T3 cells were infected with WT GETV and GETV-3ΔS2-CM1 at a MOI of 0.1. (a) Multistep growth curves. (b) Copy numbers of viral genomic RNAs in the cell lysate and (c) in supernatant were measured using RT-qPCR. (d) Relative levels of IFN-β mRNA in WT GETV and GETV-3ΔS2-CM1 infected cells were measured using RT-qPCR. Induction of IFN-β mRNA expression (fold change compared to that of GAPDH mRNA) in infected cells was calculated and obtained values were normalized to the fold changes in WT GETV infected cells (taken as 1). All experiments were performed in three biological replicates, error bars indicate standard deviation. (TIF) [file ppat.1012700.s003.tif]

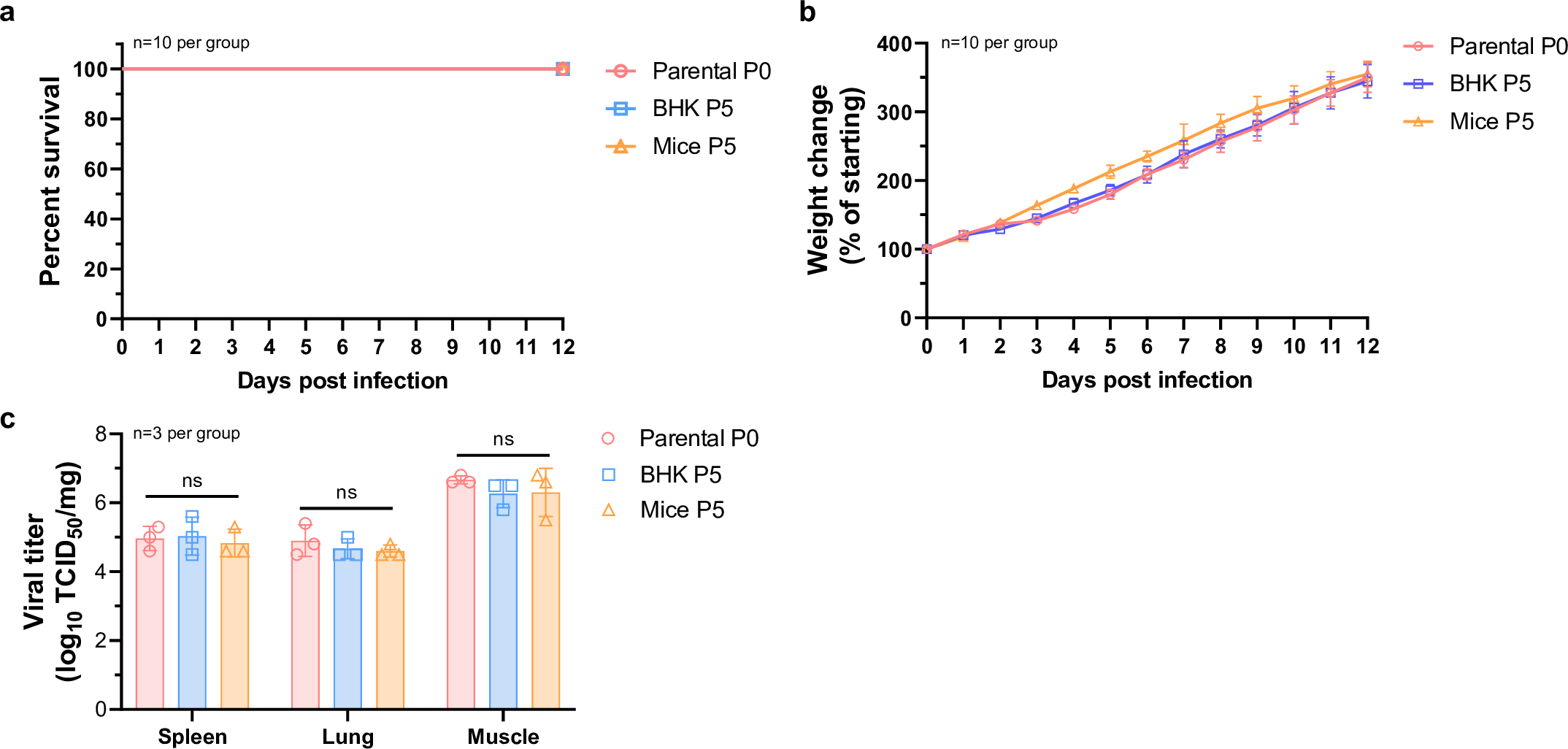

Supplement: S4 Fig — Suckling mice (n = 10 per group) were infected with a P0 stock of GETV-3ΔS2-CM1 or with viruses passaged five times in BHK-21 cells (BHK P5) or in suckling mice (Mice P5). The experiment was performed as described in the legend of Fig 1g. Survival (a) and body weight changes (b) were monitored daily. An additional group of mice (n = 3 per group) was infected with P0 stock, BHK P5 stock, or Mice P5 stock of GETV-3ΔS2-CM1. Mice were sacrificed on Day 2 post infection, and the viral loads in the spleen, lung, and muscle tissues were measured as described in the legend of Fig 1i. For panel c statistical analysis was performed using Student’s t test; ns, not significant. (TIF) [file ppat.1012700.s004.tif]

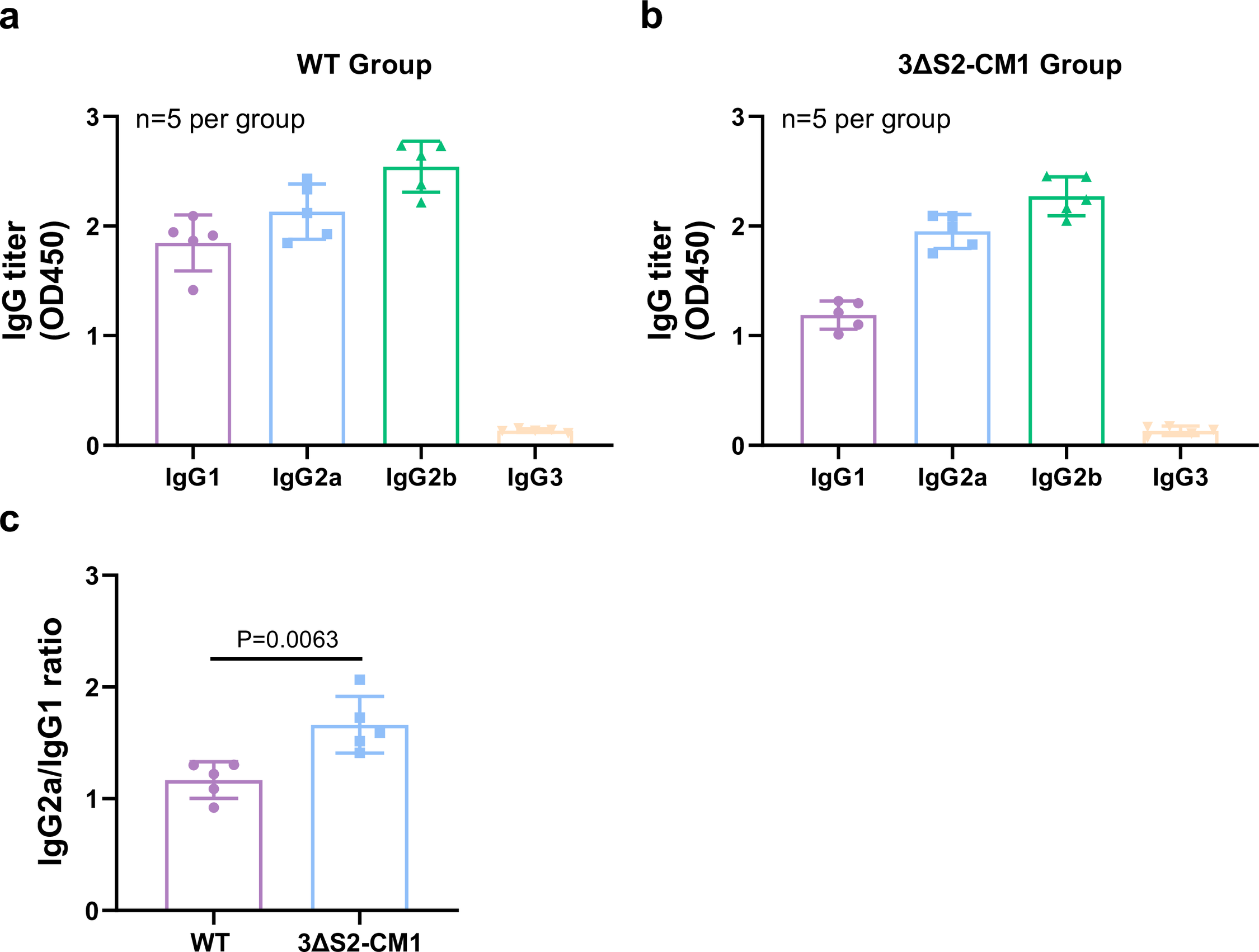

Supplement: S5 Fig — Three-week-old ICR mice (n = 5 per group) were infected with 1 × 10^5^ TCID50 of WT GETV or GETV-3ΔS2-CM1. Isotypes of GETV-specific IgG antibodies in the serum at Day 28 post infection with WT GETV (a) or GETV-3ΔS2-CM1 (b) were measured by ELISA performed using recombinant GETV p62-E1 protein; titres of IgG1, IgG2a, IgG2b and IgG3 are presented as OD450 values. (c) The ratio of IgG2a/IgG1 isotypes in sera of WT GETV and GETV-3ΔS2-CM1 infected mice. For panel c statistical analysis was performed using Student’s t test. (TIF) [file ppat.1012700.s005.tif]

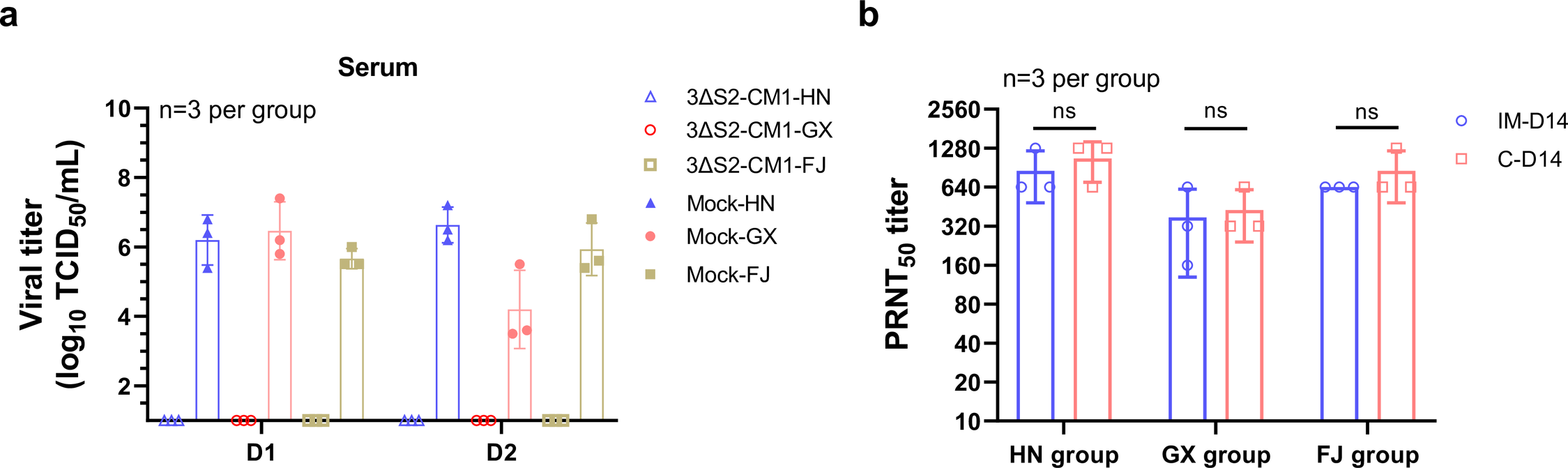

Supplement: S6 Fig — Three-week-old ICR mice (n = 9 per group) were immunized with 1 × 10^5^ TCID50 of GETV-3ΔS2-CM1 or mock-immunized with PBS. Day 14 postimmunization serum samples were collected and the mice (n = 3 per immunized and mock-immunized group) were subcutaneously challenged with 1 × 10^6^ TCID50 of GETV-HN (WT GETV), GETV-GX or GETV-FJ. (a) Viral titres in the sera of the GETV-3ΔS2-CM1 and mock-immunized mice at Day 1 and 2 post infection were determined using a TCID50 assay. (b) Neutralizing antibody titres in sera collected at Day14 postimmunization (IM-D14) and in sera collected at Day 14 post-challenge (C-D14) were measured using PRNT assay performed with matching GETV strains. Statistical analysis was performed using Student’s t test; ns, not significant. (TIF) [file ppat.1012700.s006.tif]

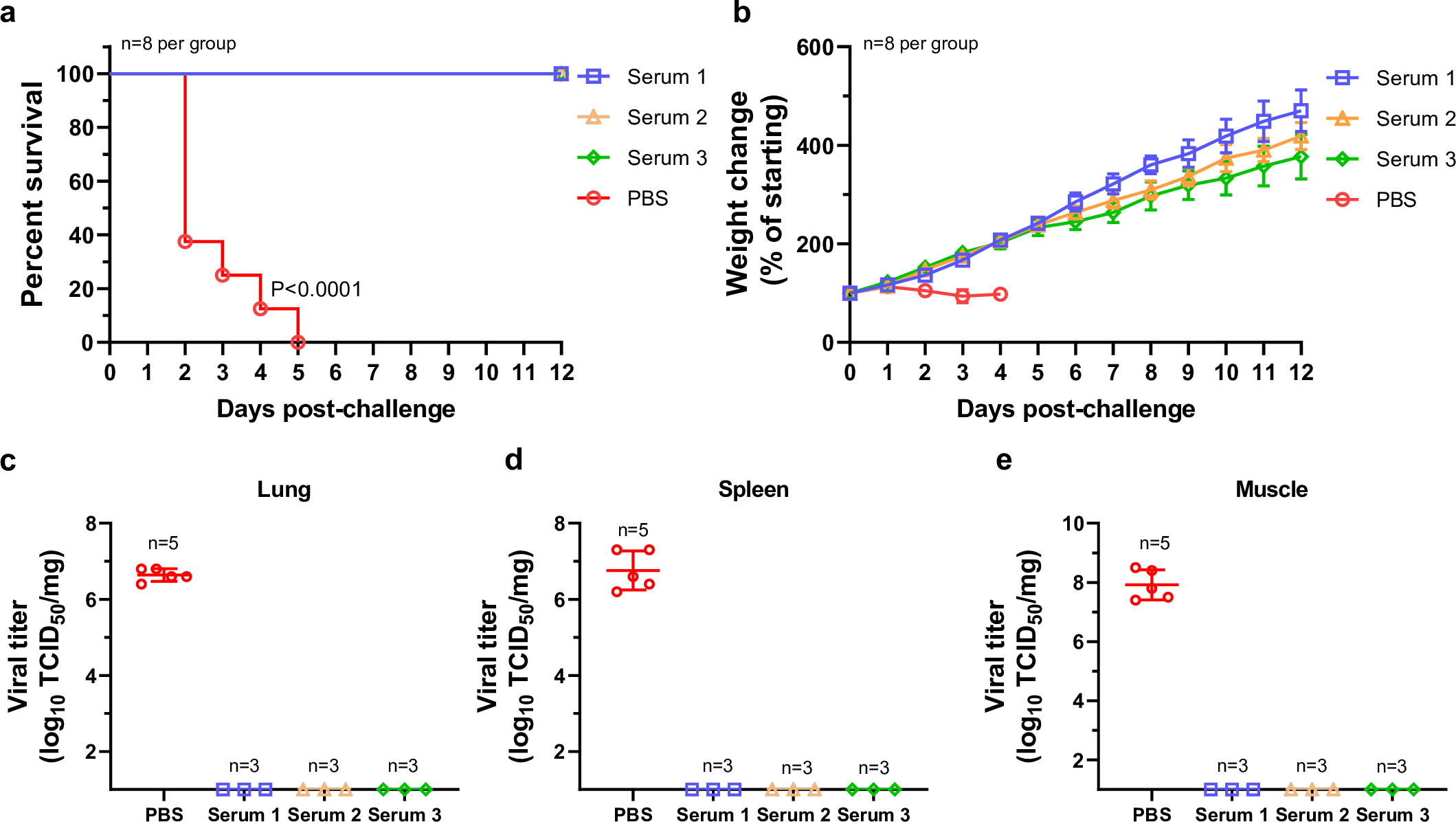

Supplement: S7 Fig — One-day-old mice (n = 8 per group) were intraperitoneally injected with 20 μL of serum collected from three GETV-3ΔS2-CM1-immunized sows on Day 28 postimmunization, and control animals were injected with PBS. Twenty-four hours later, the mice were subcutaneously challenged with 3 × 10^5^ TCID50 of WT GETV. (a) Survival and (b) body weight changes were recorded daily. (c-e) One-day-old mice (n = 5 in the PBS group and n = 3 in each the serum group) were treated as described above, challenged with WT GETV and sacrificed on Day 2 after challenge. Viral loads in the lung (c), spleen (d), and muscle (e) were determined. For panel a statistical analysis was performed using the log-rank test. P-values are shown. (TIF) [file ppat.1012700.s007.tif]

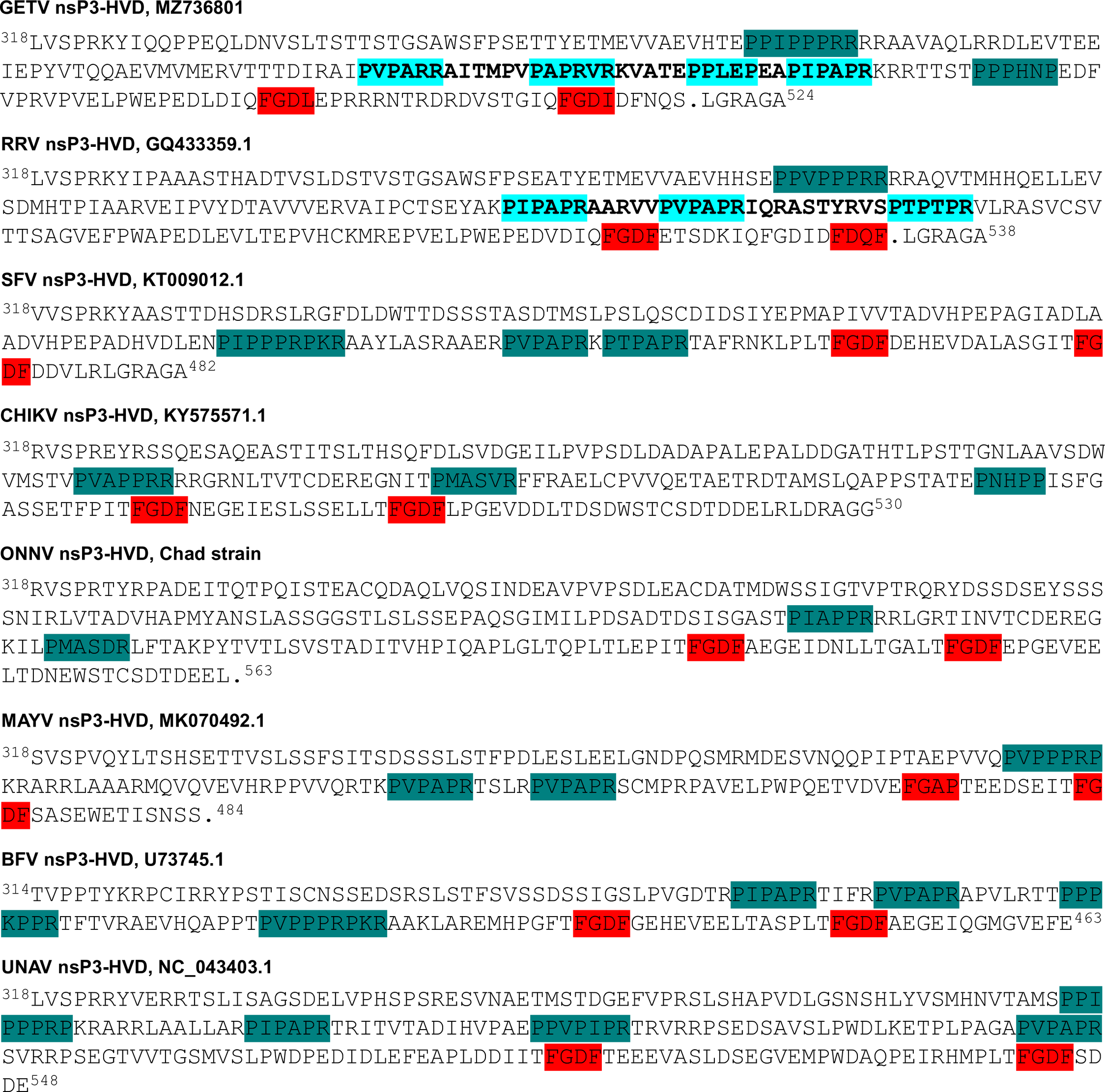

Supplement: S8 Fig — The HVDs of nsP3 of alphaviruses have low sequence similarity and cannot be reliably aligned. Instead, they contain short sequence motifs used to interact with host proteins. G3BP binding motifs crucial for the replication of Old World alphaviruses are annotated in red. In addition, the HVDs of nsP3 in GETV, RRV, SFV, CHIKV, ONNV, MAYV, BFV and UNAV contain multiple proline-rich motifs annotated in dark cyan. Residues deleted in GETV-3ΔS2-CM1 and RRV-3ΔS2-CM1 are shown in bold, and the four (GETV) or three (RRV) proline-rich motifs, removed by deletions, are annotated in blue. Positions of the first and the last residue in the corresponding protein are indicated. (TIF) [file ppat.1012700.s008.tif]

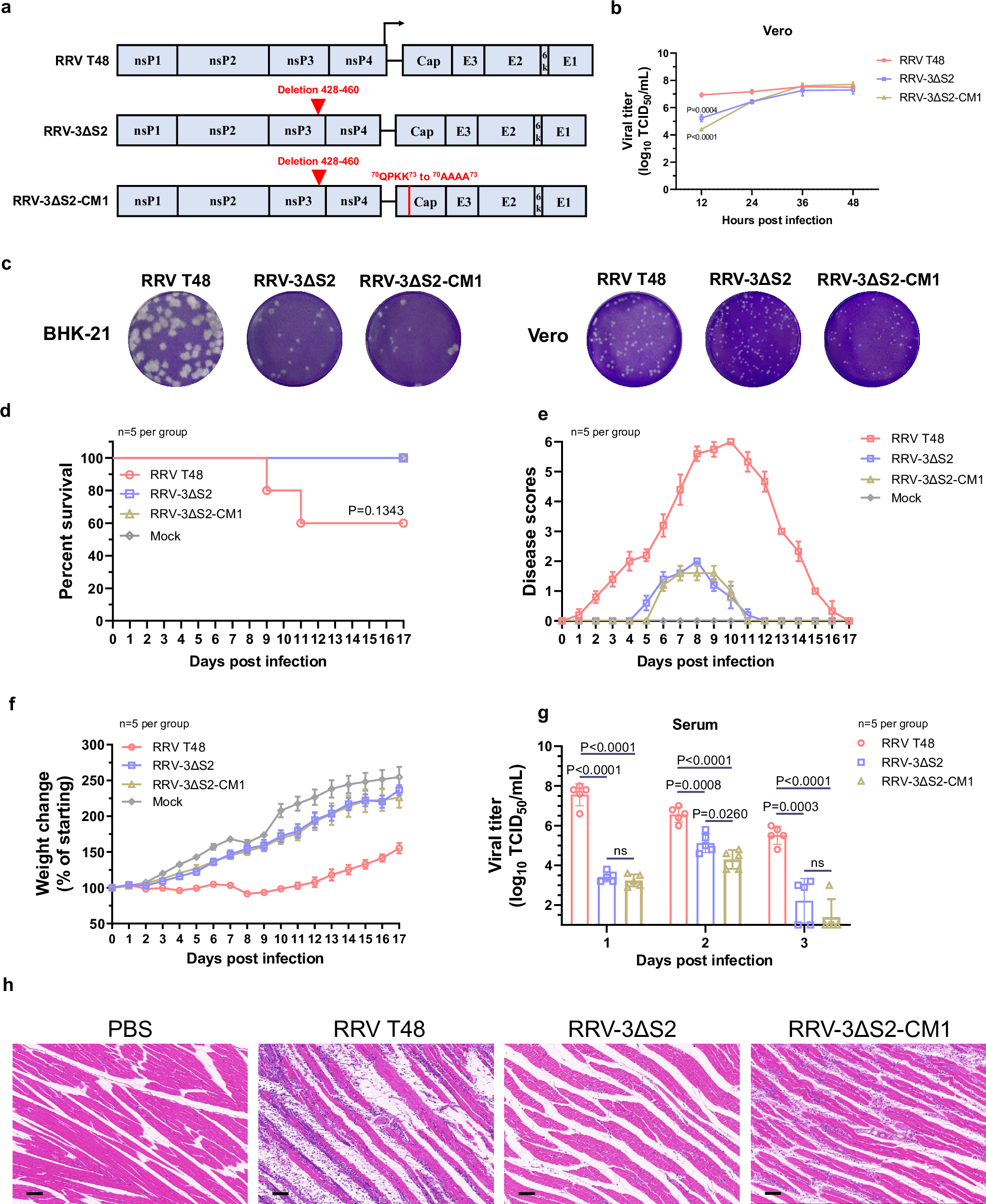

Supplement: S9 Fig — (a) Schematic representation of the genomes of RRV T48, RRV-3ΔS2, and RRV-3ΔS2-CM1; the mutations are marked as in Fig 1a. (b) Multistep growth curves (MOI of 0.1) of RRV T48, RRV-3ΔS2, and RRV-3ΔS2-CM1 in Vero cells. P values indicate statistical significance of differences between titres of RRV T48 and its mutant variants at 12 hpi. (c) Plaque morphologies of RRV T48, RRV-3ΔS2, and RRV-3ΔS2-CM1 on BHK-21 and Vero cells. (d-f) Seventeen-day-old ICR mice (n = 5 per group) were subcutaneously inoculated with 1 × 10^4^ TCID50 of RRV T48, RRV-3ΔS2, RRV-3ΔS2-CM1 or mock-inoculated with PBS. Survival (d), disease score (e), and weight change (f) were monitored daily. (g) Viral titres in serum samples from RRV T48-, RRV-3ΔS2-, and RRV-3ΔS2-CM1-infected mice (n = 5 per group) at Days 1, 2 and 3 post infection. (h) H&E staining of the quadriceps femoris muscle from mice infected with RRV T48, RRV-3ΔS2, or RRV-3ΔS2-CM1 or mock-infected with PBS at 10 days post infection. Representative images are shown, (scale bar 100 μm). For panels b and g statistical analysis was performed using Student’s t test. For panel d statistical analysis was performed using the log-rank test. P-values are shown; ns, not significant. (TIF) [file ppat.1012700.s009.tif]

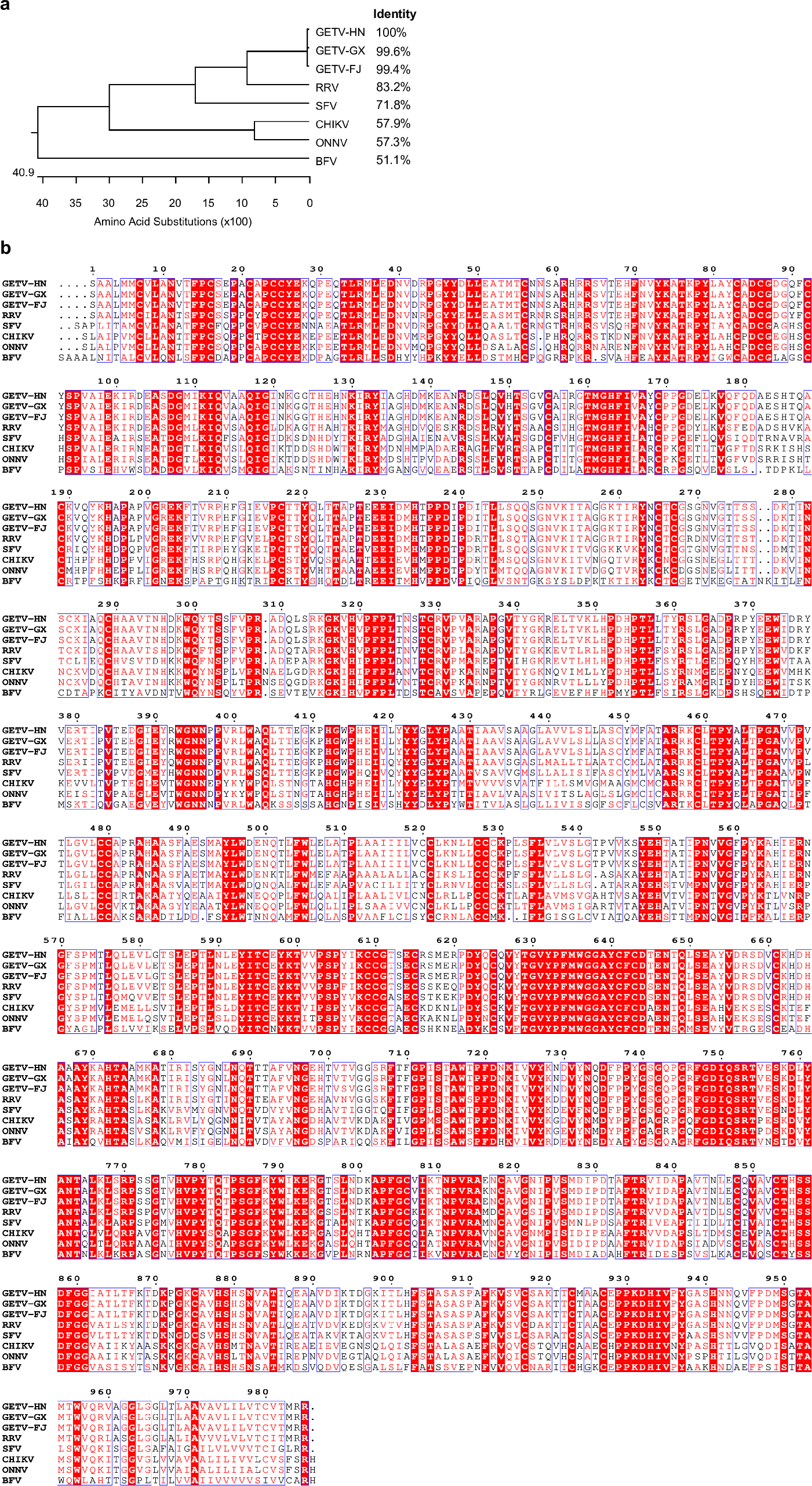

Supplement: S10 Fig — (a) Phylogenetic tree constructed using Lasgene7.1 software. Sequence alignment was performed by the Clustal W method. The percentage of amino acid sequence identity between GETV-HN (WT GETV) and other viruses is presented on the right. (b) Alignment of E3-E2-6K-E1 protein sequences of GETV-HN, GETV-GX, GETV-FJ, RRV, SFV, CHIKV, ONNV, and BFV. Amino acid residues are numbered according to the residues in the E3-E2-6K-E1 of GETV-HN. (TIF) [file ppat.1012700.s010.tif]
